# Lessons drawn from Shanghai for controlling highly transmissible SARS-CoV-2 variants: insights from a modelling study

**DOI:** 10.1186/s12879-023-08316-7

**Published:** 2023-05-16

**Authors:** Hao Wang, Tangjuan Li, Huan Gao, Chenxi Huang, Biao Tang, Sanyi Tang, Robert A. Cheke, Weike Zhou

**Affiliations:** 1 School of Mathematics and Statistics, Shaanxi Normal University, Xi’an, 710062 PR China; 2 School of Mathematics and Statistics, Xi’an Jiaotong University, Xi’an, 710049 PR China; 3 Natural Resources Institute, University of Greenwich at Medway, Central Avenue, Chatham Maritime, Kent, ME4 4TB UK; 4 School of Mathematics, Northwest University, Xi’an, 710127 PR China

**Keywords:** COVID-19, Step-by-step NPI release strategy, City-based pattern, District-based pattern, Optimal control

## Abstract

**Background:**

The continuous emergence of novel SARS-CoV-2 variants with markedly increased transmissibility presents major challenges to the zero-COVID policy in China. It is critical to adjust aspects of the policy about non-pharmaceutical interventions (NPIs) by searching for and implementing more effective ways. We use a mathematical model to mimic the epidemic pattern of the Omicron variant in Shanghai to quantitatively show the control challenges and investigate the feasibility of different control patterns in avoiding other epidemic waves.

**Methods:**

We initially construct a dynamic model with a core step-by-step release strategy to reveal its role in controlling the spread of COVID-19, including the city-based pattern and the district-based pattern. We used the least squares method and real reported case data to fit the model for Shanghai and its 16 districts, respectively. Optimal control theory was utilized to explore the quantitative and optimal solutions of the time-varying control strength (i.e., contact rate) to suppress the highly transmissible SARS-CoV-2 variants.

**Results:**

The necessary period for reaching the zero-COVID goal can be nearly 4 months, and the final epidemic size was 629,625 (95%CI: [608,049, 651,201]). By adopting the city-based pattern, 7 out of 16 strategies released the NPIs more or earlier than the baseline and ensured a zero-resurgence risk at the average cost of 10 to 129 more cases in June. By adopting the district-based pattern, a regional linked release can allow resumption of social activity to ~ 100% in the boundary-region group about 14 days earlier and allow people to flow between different districts without causing infection resurgence. Optimal solutions of the contact rate were obtained with various testing intensities, and higher diagnosis rate correlated with higher optimal contact rate while the number of daily reported cases remained almost unchanged.

**Conclusions:**

Shanghai could have been bolder and more flexible in unleashing social activity than they did. The boundary-region group should be relaxed earlier and more attention should be paid to the centre-region group. With a more intensive testing strategy, people could return to normal life as much as possible but still ensure the epidemic was maintained at a relatively low level.

**Supplementary Information:**

The online version contains supplementary material available at 10.1186/s12879-023-08316-7.

## Introduction

Since the first SARS-CoV-2 case reported at the end of December 2019 [1, 2], the COVID-19 pandemic has lasted for almost three years, which poses major threats to human society. To combat the COVID-19 pandemic, a toolkit of non-pharmaceutical interventions (NPIs) has been developed worldwide, including wearing masks, social distancing, personal hygiene, testing, tracing and isolating infected people to effectively cut off the source of infection, etc. [3]. Control interventions tailored to national circumstances are urgently needed, with the main objective of designing appropriate or optimal intervention implementation strategies in the most cost-effective manner to control the spread of SARS-CoV-2. Many countries have made progress in this regard over the past three years [4–6]. For example, from the outset of the pandemic until May 2022, China has experienced dozens of local outbreaks of original, Delta and Omicron variants [7–15].

However, as new variants continuously emerge, the transmission ability of SARS-CoV-2 is consistently increasing [16–20]. For example, the Delta variant first discovered in India is 97% more transmissible than the original strain [21], and the effective (instantaneous) reproduction number of Omicron is 3.19 times greater than that of Delta under the same epidemiological conditions [22]. Note that the vaccination programme has been actively promoted and boosters have been administered against the COVID-19 infection, thereby reducing the severity of infections, numbers of hospitalizations and deaths [23–27]. However, the long-term effectiveness of the vaccine is still unknown, as breakthrough infections [28–31] indicate a potential risk of illness after successful vaccination and subsequent infection with a different variant of the virus [31]. As a result, outbreaks are becoming more frequent and widespread in mainland China. Most recently, the ongoing epidemic in Shanghai, China, reached a peak number of daily reported cases of 27,605 on 13 April 2022 [32], which breaks the high number records of all of the former local outbreaks in mainland China [33, 34]. This brings new challenges to the established control policies, which urgently require dynamic adjustments to their strategies.

The zero-COVID policy’s principle is to use aggressive interventions to control the cases down to zero in a short period. The outcomes of China’s multiple indigenous outbreaks mentioned above strongly support the case for the dynamic zero-COVID policy in China. However, the highly increased transmission ability of Omicron variants is continuously challenging the tolerable controlling period and costs leading to the need to alter current policies. The local outbreak in Shanghai in March 2022 showed different characteristics from previous local outbreaks in China. For example, in the early stage of the epidemic, the intensity of the government’s control measures was gradually increased rather than imposing a one-time adjustment. After the epidemic was brought under control, the government again slowly and gradually released the control interventions rather than all at once, which was called the step-by-step release strategy. We named it the city-based pattern control by regarding Shanghai as a whole. In addition, note that the outbreak size and epidemic duration in different districts of Shanghai varied significantly, and they seemed to correlate with the distribution of population density and geographical location. Is there a strategy to release control interventions in advance in those areas where the outbreak was contained earlier? We try to explore a region-specific release strategy, which we term a district-based pattern.

To quantitatively explore feasible ways to maintain epidemics at low levels for the variants with high transmissibility is the subject of this study. We use a mathematical model to mimic the epidemic pattern of Omicron under the control strength in China to quantitatively show the challenges in the control of the outbreak of Omicron, and then to investigate the feasibility of various combinations of the gradual and region-dependent releases of control interventions in avoiding other epidemic waves. In addition, we model the effects of dynamically changing intervention intensity using optimal control theory, which refers to the problem of seeking an optimal amount of control to minimize a certain performance indicator for a controlled system under certain constraints. Finally, we also conduct a retrospective analysis of the timing of the implementation of control interventions in Shanghai from a city-based pattern.

## Methods

### Model overview

**Fig. 1:**
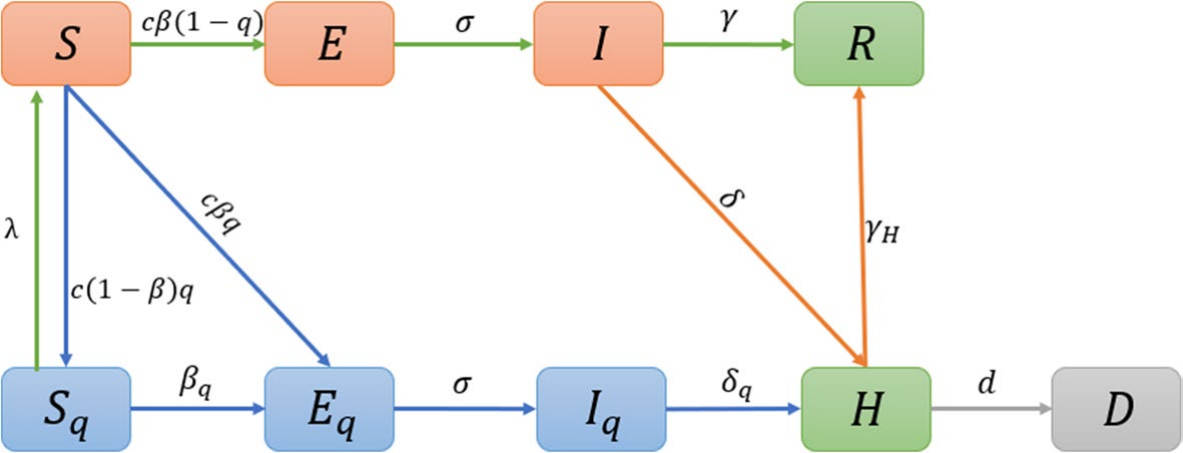
Schematic diagram illustrating the COVID-19 transmission in the local outbreak in Shanghai city, China

Based on a Susceptible- Exposed- Infectious- Isolated and Hospitalized- Recovered model structure (SEIHR) [35–37], we developed a dynamic model of COVID-19 transmission in the local outbreak caused by the SARS-CoV-2 Omicron variant in Shanghai, as shown in model (1) and the flow diagram in Fig. 1. According to different physical activity levels, the population not being hospitalized can be divided into two subgroups: the free population and the quarantined population (labelled with subscript ‘q’). The former represents the people under closed-loop management, while the latter represents other relevant risk groups [38]. We assumed that there was no cross infection between the two subgroups. The free population was divided into susceptible ($S$), exposed ($E$), infectious ($I$), and recovered ($R$) classes. The transmission occurs when the susceptible population become close contacts of infectious people and become infected with a transmission probability $\beta$ for each contact. In addition, given the implementation of a close-contact tracing strategy, we assumed that a proportion $q$ of the close contacts can be traced and quarantined, moving to the quarantined susceptible class (${S}_{q}$) or quarantined exposed class (${E}_{q}$) according to whether they are infected or not. The untraced population (a proportion 1$-q$ of the close contacts) will stay in the susceptible class ($S$) if they are not infected (with probability 1-$\beta$) or move to the exposed class ($E$) if they are infected (with transmission probability $\beta$). Individuals in exposed ($E$) /quarantined exposed (${E}_{q}$) class will enter into the infectious ($I$) /quarantined infectious (${I}_{q}$) class with the transition rate $\sigma$, where $1/\sigma$ is the incubation period. Further, the infectious/ quarantined infectious individuals will be isolated and hospitalized after being diagnosed, entering into the class $H$. Note that in China people testing positive for COVID-19 are hospitalized, unlike in other countries where this is only the case for those who are seriously ill and where transitions from ${I}_{q}$ to $H$ are common. Considering the fact that there may exist transmission or new infections amongst the quarantined population, we assumed a transmission rate ${\beta }_{q}$ of COVID-19 from quarantined infectious individuals to quarantined susceptible individuals, which actually equals the contact rate among quarantined population times the transmission probability per contact.

In model (1), $c\left(t\right)$ is the contact rate, $\lambda$ is the release rate of the quarantined susceptible individuals, $\delta \left(t\right)$, ${\delta }_{q}$ are the diagnosis rates for individuals in compartments $I$ and ${I}_{q}$, respectively. $\gamma$ and ${\gamma }_{H}$ are the natural recovery rates for individuals in classes $I$ and $H$, respectively. A disease-related death for the hospitalized population is considered with rate $d$. The compartment D represents the dead class. A more detailed description of the parameters and variables is given in Table 1.

1 $$\begin{array}{l}\frac{dS}{dt}=\lambda S_q-c\left(t\right)\beta\frac{SI}{S+E+I\text{+}R}-c\left(t\right)\left(1-\beta\right)q\left(t\right)\frac{SI}{S+E+I+R}\\\frac{dE}{dt}=c\left(t\right)\beta\left(1-q\left(t\right)\right)\frac{SI}{S+E+I+R}-\sigma E\\\frac{dI}{dt}=\sigma E-\gamma I-\delta\left(t\right)I\\\frac{dS_q}{dt}=c\left(t\right)\left(1-\beta\right)q\left(t\right)\frac{SI}{S+E+I+R}-\beta_q\frac{S_qI_q}{S_q+E_q+I_q}-\lambda S_q\\\frac{dE_q}{dt}=c\left(t\right)\beta q\left(t\right)\frac{SI}{S+E+I+R}+\beta_q\frac{S_qI_q}{S_q+E_q+I_q}-\sigma E_q\\\frac{dI_q}{dt}=\sigma E_q-\delta_qI_q\\\frac{dH}{dt}=\delta\left(t\right)I+\delta_qI_q-dH-\gamma_HH\\\frac{dR}{dt}=\gamma I+\gamma_HH\\\frac{dD}{dt}=dH\end{array}$$

Considering that the package of NPIs was dynamically changing as it was following the development of the epidemics, the control-related parameters (the contact rate c(t), the diagnosis rate $\delta \left(t\right)$ and the quarantine rate $q\left(t\right)$ were assumed to be time-dependent according to the implementation schedule of control interventions listed in the Supplementary material [38]. Particularly, the contact rate $\text{c}\left(\text{t}\right)$ was given as follows.

2 $$c\left(t\right)=\left\{\begin{array}{ccc}c_1,&0\leq t<t_1,&\mathrm{before}\;2\;\mathrm{April},\\c_2,&t_1\leq t\leq t_2,&2-10\;\mathrm{April},\\c_3,&t_2<t\leq t_3,&11-18\;\mathrm{April},\\c_4,&t_3<t\leq t_4,&19-28\;\mathrm{April},\\k(t-t_4)+c_4,&t_4<t\leq t_5,&29\;\;\mathrm{April}\;\mathrm{to}\;31\;\mathrm{May},\end{array}\right.$$

where ${t}_{1}=33, {t}_{2}=41,{t}_{3}=49,{t}_{4}=59, {t}_{5}=92$ by taking 1 March 2022 as the initial time. Note that the control interventions were dynamically adjusted to be relaxed partially after 28 April 2022 when the contact rate was assumed to follow a linear increasing function when $t>{t}_{4}$. The diagnosis rate $\delta \left(t\right)$ and quarantine rate $q\left(t\right)$ were set to be piecewise constant functions in three phases, as defined in Table 1.

**Fig. 2:**
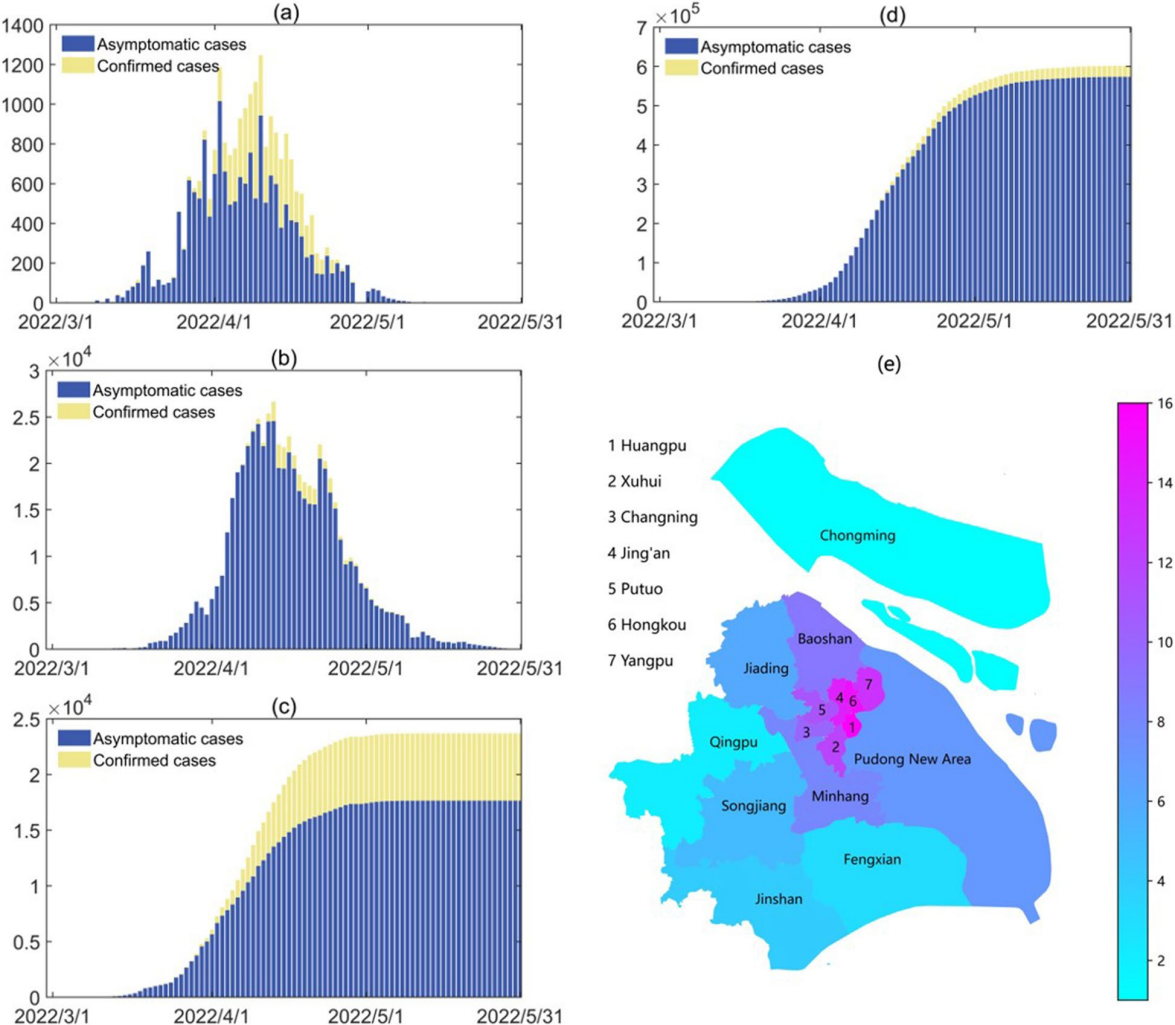
**a**-**b** The daily reported cases between 1 March and 31 May 2022 from **a** the non-quarantined population and **b** the quarantined population in Shanghai. **c**-**d** The accumulative reported cases between 1 March and 31 May 2022 from **c** the non-quarantined population and **d** the quarantined population in Shanghai. **e** Distribution of the 16 districts in Shanghai city. The population density was ranked from low to high, represented by different colors, as shown in the color bar on the right with values varying from 1 to 16

Shanghai has 16 districts (Fig. 2(e)), we assumed that there was no movement of people between any two districts, and the model framework describing the COVID-19 transmission dynamics in each district was the same as that in Shanghai.

### Data

Multi-source epidemic data of COVID-19 related to the local outbreak caused by the SARS-CoV-2 Omicron variant in Shanghai were obtained from the Shanghai Municipal Health Commission [33]. The data are public and easily available on the website [33, 34, 38]. Shanghai Municipal government announced the start of the epidemic by holding a press conference on epidemic prevention and control on March 1, 2022, and officially announced a major change in the epidemic control policy on June 1, so we chose this period to fit the model. The data information included a time series of the number of daily reported cases between 1 and 2022 and 31 May 2022, which was further separated into two groups: newly reported cases from the non-quarantined population and newly reported cases from the quarantined population, as shown in Fig. 2(a-d). Note that the newly reported cases included both the newly reported COVID-19 positive but asymptomatic cases and the newly reported confirmed symptomatic cases (excluding those transferred from the asymptomatic cases). In obtaining and processing the data, we found that the above COVID-19 epidemic data could be further divided into 16 groups according to the respective residential district of the reported cases. And the sizes of the outbreak and duration of the epidemic in these 16 districts are quite different. Furthermore, these seemed to be related to population density distributions and geographical locations. Therefore, 16 districts in Shanghai were also considered for in-depth analysis. The data were released and analyzed anonymously.

### Model fitting process

The data of COVID-19 in Shanghai and the 16 districts between 1 and 2022 to 31 May 2022 were fitted to model (1), respectively, to calibrate the model. We firstly fixed some parameters in model (1) by reviewing literature, which included the incubation period ($1/\sigma$), the release rate of quarantined susceptible individuals ($\lambda$), the natural recovery rate of infected individuals (${\upgamma }$) and the recovery rate of hospitalized individuals (${\gamma }_{H}$). The initial susceptible population was fixed as the total population in Shanghai and the corresponding district, respectively. The initial infected quarantined susceptible/exposed/infected population hospitalized population, recovered and dead populations were obtained from the database, as shown in Tables 1 and S1-S4.

Next, unknown parameters were estimated by using the nonlinear least squares method to fit the model to the time series data (between 1 March to 31 May 2022) of newly reported cases from the non-quarantined population and newly reported cases from the quarantined population in Shanghai and the 16 districts. One hundred groups of parameter estimations were obtained by perturbing the pre-set values of unknown parameters which were assumed to follow the uniform distribution. The nonlinear least-squares method was carried out in MATLAB, using the “ODE45” function to solve the ODE system and the “fmincon” function to search for optimal solutions of the objective function.

### Optimal control problem

We utilized optimal control theory to explore the solution of the dynamic control strength to maintain the epidemic below a targeted level. To this end, we formulated an Optimal Control Problem (OCP) to find an optimal (time-varying) reduction rate of the contact rate to minimize the cost produced by the prevention and control interventions while ensuring that the number of newly reported cases is always less than the pre-set threshold. The detailed formulation is provided in the Supplementary material.

We aimed to maintain the number of daily reported cases below the targeted number in a finite period $T$, which was named the control duration. Thus, it is reasonable to assume that the optimal control program would be initialized when the epidemic was controlled to a low level, hence a threshold condition which triggered the optimal control program was given. The simulations of the optimal control problem were completed in MATLAB with the help of OpenOCL- Open optimal control library [39].

## Results

### Baseline estimation and prediction

**Fig. 3:**
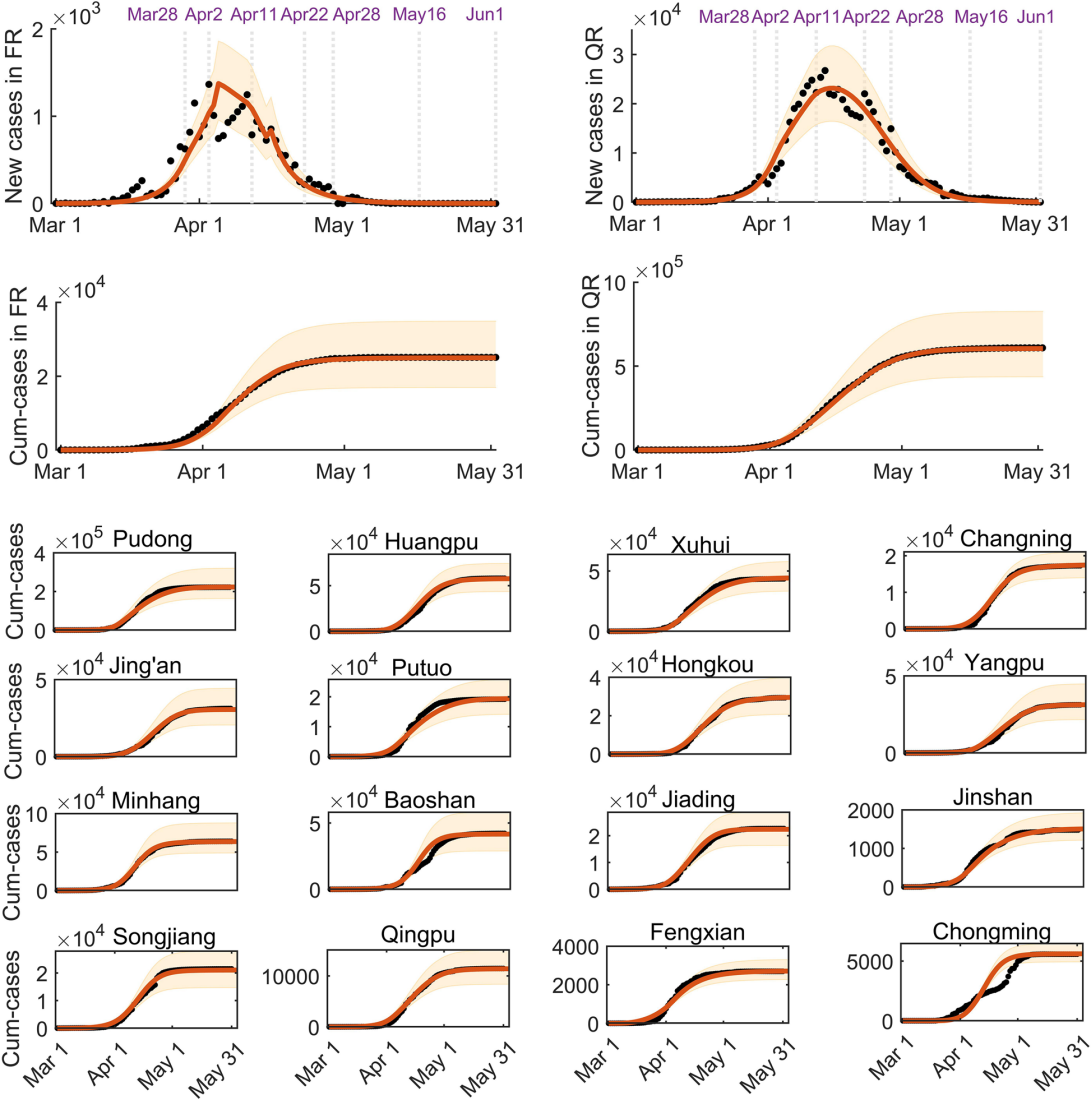
Fitting results of model (1) to the epidemic data in Shanghai and to the 16 districts, respectively. The two graphs in the first/ second row show the fitting of newly reported cases/ cumulative number of reported cases from the non-quarantined population (left) and the quarantined population (right) in Shanghai, respectively. The rest of the graphs show the fitting of the cumulative number of reported cases in the 16 districts, respectively. The black points represent the reported epidemic data, the solid red curves with the shadow areas are the best fitting curves and their 95% confidence intervals

The best fitting results of the model to the epidemic data of Shanghai city are shown in Fig. 3, correspondingly the estimated values of the unknown parameters and initial conditions are listed in Table 1. The results showed that the number of newly reported cases in Shanghai had fallen below 30 on 31 May 2022, and we predicted that the zero-COVID goal would be reached on 24 June (95% CI: 18 June to 1 July), and the final epidemic size would be 629,625 (95%CI: [608,049, 651,201]) if the NPIs’ intensity after 31 May 2022 was maintained as the same as it was on 31 May 2022. This is consistent with the result in [40], whose estimate is about 626,000. The effective reproduction number was calculated by using the renewal equation method (see details in the Supplementary material). In addition, the fitting results of the model to the respective accumulative number of reported cases in the 16 districts are shown in Fig. 3 and the parameter estimation results are listed in Tables S1-S4. In addition, we obtained the time when the number of daily reported cases fell below 5 in each district, which correlated with the population density in the corresponding district, as shown in Table S5 in the Supplementary material [41]. The results showed that the daily reported number of cases in 6 of the 16 districts had fallen to 5 before 24 May (on 17 May on average). These districts, Jiading, Songjiang, Jinshan, Fengxian, Qingpu and Chongming, were just those districts located on the boundary of Shanghai city (Fig. 2(e)) with the population density less than 2000 persons/km^2^ (Table S5). Meanwhile, the daily number of reported cases fell to 5 after 26 May (on 5 June on average), in the other 10 districts whose population densities were more than 2000 persons/ km^2^, much later than those in districts with smaller population densities.

The baseline estimation and prediction showed that, although the number of newly reported cases in Shanghai had fallen below 30 on 31 May 2022, the time required to reach the zero-COVID goal would be almost 1 month even with the same intensity of NPIs as on 31 May 2022. The Shanghai government had announced that it would unlock the city from 1 June to deal with the subsequent epidemic trend. Therefore, in the next section, we investigate how different unlocking strategies / release strategies of the control interventions (including release patterns and intensities) will affect the prevention and control of the epidemic in Shanghai and the main epidemic trend.

**Table 1:** Definition and values of parameters and variables in model (1) in the local outbreak in Shanghai

**Parameter**		**Definition**	**Value(95%CI)**	**Source**
$c$	--	Contact rate among free population	--	--
	${c}_{1}$	Contact rate before 2 April	31.895[31.072–32.732]	Estimated
	${c}_{2}$	Contact rate from 3 to 10 April	16.460[15.939–17.011]	Estimated
	${c}_{3}$	Contact rate from 11 to 18 April	6.823[6.659–6.936]	Estimated
	${c}_{4}$	Contact rate from 19 to 28 April	8.212[8.012–8.387]	Estimated
	$k$	Linear increasing rate of contact rate from 28 April to 31 May	0.150[0.147–0.154]	Estimated
$\beta$		Transmission probability per contact among the non-quarantined population	0.085[0.082–0.087]	Estimated
${\beta }_{q}$		Transmission rate from quarantined infectious to quarantined susceptible individuals	0.806[0.783–0.824]	Estimated
$\lambda$		Release rate of quarantined susceptible individuals	1/14	[38]
$\sigma$		Transition rate from exposed to infectious	1/3	[42]
$q$		Tracing and quarantine rate	--	--
	${q}_{1}$	Quarantine rate before 28 March	0.618[0.604–0.629]	Estimated
	${q}_{2}$	Quarantine rate from 29 March to 14 April	0.757[0.734–0.769]	Estimated
	${q}_{3}$	Quarantine rate from 15 April	0.799[0.779–0.817]	Estimated
$\delta$		Diagnosis rate of infectious individuals	--	--
	${\delta }_{1}$	Diagnosis rate before 3 April	0.221[0.214–0.228]	Estimated
	${\delta }_{2}$	Diagnosis rate from 4 to 14 April (Nucleic acid testing once every two days with antigen testing)	0.273[0.267–0.279]	Estimated
	${\delta }_{3}$	Diagnosis rate from 15 April (Nucleic acid testing once per day with antigen testing)	0.374[0.366–0.382]	Estimated
${\delta }_{q}$		Diagnosis rate of quarantined infectious individuals	0.642[0.622–0.659]	Estimated
$\gamma$		Natural recovery rate of infectious individuals	1/7	[38]
${\gamma }_{H}$		Recovery rate of hospitalized and isolated cases	1/7	[38]
$d$		Disease-related death rate	2.995[2.888–3.079]*e-5	Estimated
**Initial value**		**Definition**	**Value(95%CI)**	**Source**
$S\left(0\right)$		Initial susceptible population	24,900,000	Data
$E\left(0\right)$		Initial exposed population	10.535[10.335–10.794]	Estimated
$I\left(0\right)$		Initial infectious population	1	Data
${S}_{q}\left(0\right)$		Initial quarantined susceptible population	277	Data
${E}_{q}\left(0\right)$		Initial quarantined exposed population	0	Assumed
${I}_{q}\left(0\right)$		Initial quarantined infectious population	1	Data
$H\left(0\right)$		Initial hospitalized and isolated population	0	Data
$R\left(0\right)$		Initial recovered population	0	Data
$D\left(0\right)$		Initial dead population	0	Data

### Feasibility of various strategies centred on the step-by-step release of NPIs

In this section, we discussed the feasibility of various release strategies of Shanghai city regarding the resurgence risk and cumulative number of reported cases in June. Given that Shanghai had gradually relaxed the control interventions from 28 April onwards, NPIs were assumed to be gradually relaxed after 28 April, which is reflected in the non-decreasing of the contact rate. In terms of time, we divide the relaxation into two or three phases. For the two-phase strategy, it is composed of a continuous release process and a process with unchanged control interventions. The contact rate $c\left(t\right)$ is consequently assumed to be a piecewise function composed of a linear growth function and a constant function, as shown in Eq. (3). Correspondingly, the contact rate in the three-phase strategy was assumed to be composed of two linear growth functions with different growth rates, as shown in Eq. (4).

3 $$c\left(t\right)=\left\{\begin{array}{c}{\phi }_{k}k\left(t-{t}_{4}\right)+{c}_{4}, { t}_{4}<t<{t}_{N},\\ c\left({t}_{N}\right), t\ge {t}_{N}. \end{array}\right.$$

4 $$c\left(t\right)=\left\{\begin{array}{c}\begin{array}{c}{\phi }_{k}^{1}k\left(t-{t}_{4}\right)+{c}_{4}, { t}_{4}<t<{t}_{N}^{1},\\ {\phi }_{k}^{2}k\left(t-{t}_{4}\right)+{c}_{4}, {t}_{N}^{1}<t<{t}_{N}^{2},\end{array}\\ \begin{array}{c} \\ c\left({t}_{N}\right), t\ge {t}_{N}^{2}.\end{array} \end{array}\right.$$

**Fig. 4:**
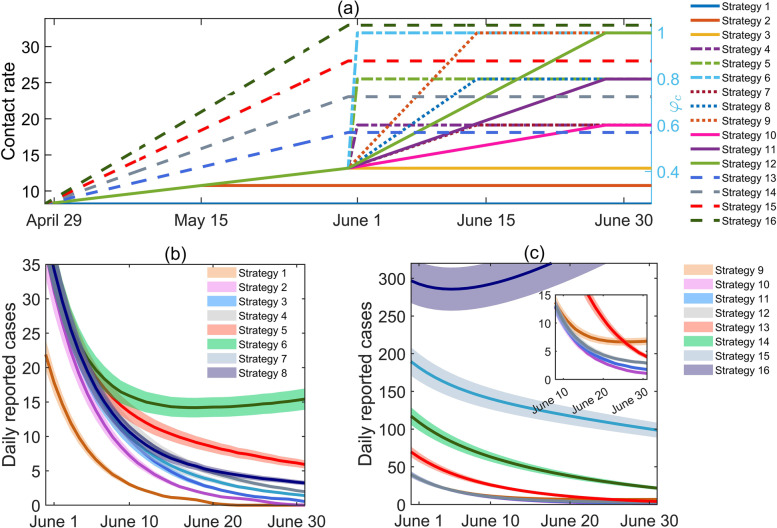
Changes in contact rates in 16 strategies after 28 April and the corresponding curves of the numbers of daily reported cases in June. **a** Time-varying contact rate in 16 strategies. Different combinations of colors and line types were used to represent different strategies. **b** Time-varying daily reported numbers of cases by adopting strategies 1–8. **c** Time-varying daily numbers of reported cases by adopting strategies 9–16. Shadows represented a 10% random perturbation of the simulation results

In the above equations, ${\phi }_{k}$ or ${\phi }_{k}^{1}$, ${\phi }_{k}^{2}$ were the modification factors of the linear growth rate with respect to the baseline increasing rate $k$, ${t}_{N}$ or ${t}_{N}^{2}$ was the corresponding time when NPIs were swiftly released, ${t}_{N}^{1}$ was the corresponding time when the linear growth phase switched, $c\left({t}_{N}\right)$ represents the maximum social activity level after the relaxing strategy. Define ${\phi }_{c}=\frac{c\left({t}_{N}\right)}{{c}_{1}}$, which represents the ratio of the resumed social activity level to the normal social activity level in pre-endemic duration, reflecting the ultimate relaxation degree. Then 16 relaxation strategies were given with different combinations of ${\phi }_{c}$, ${\phi }_{k}$ (${\phi }_{k}^{2}$) and ${t}_{N}$ (${t}_{N}^{2}$). In fact, strategies 1–6, 13–16 belong to the two-phase strategy, while strategies 7–12 belong to the three-phase strategy, as shown in Fig. 4(a) and Table 2. Figure 4 shows the time-varying contact rate $c\left(t\right)$ after 28 April in each strategy and the corresponding time-varying daily reported cases between 1 and 30 June. Table 2 lists the combination of each strategy and the resurgence risk ($P$) and the cumulative reported number of cases (${I}_{sum}$) between 1 and 30 June. In the 16 strategies, strategy 3 was the baseline estimation scenario.

The results showed that strategies 4, 5, 7, 8, 10, 11, 12 would be more preferred choices, which would not trigger a resurgence or lead to significantly increased numbers of reported cases (see Table 2) while social activity could be resumed to higher levels (greater ${\phi }_{c}$ than the baseline scenario) without delaying the relaxation timing. The relaxation strategy adopted by the Shanghai government in reality was to swiftly relax NPIs on 1 June, which led to 304 reported cases in June. This is closest to the relaxation strategy 4, leading to 306 (95%CI: [287–324]) reported cases in June. However, an intense relaxation strategy of NPIs, for instance, strategies 6 or 9, which completely relaxed the NPIs too early (resuming to the normal social activity quickly with ${\phi }_{c}=1$) would lead to a high resurgence risk of 53% or 18%, with corresponding ${I}_{sum}$ being 514 (95%CI: [481–547]) or 350 (95%CI: [328–371]) respectively. Worse still, accelerating the relaxation strategy between 28 and 28 and 31 May would significantly cause more infections in June (strategy 13–16). Particularly, in strategy 16, the NPIs were acceleratively relaxed after 28 April and social activity was completely resumed to the normal condition on 31 May, so the COVID-19 outbreak in Shanghai would flare up quickly due to the high transmissibility of the Omicron variant and the continued existence of an infectious source.

**Fig. 5:**
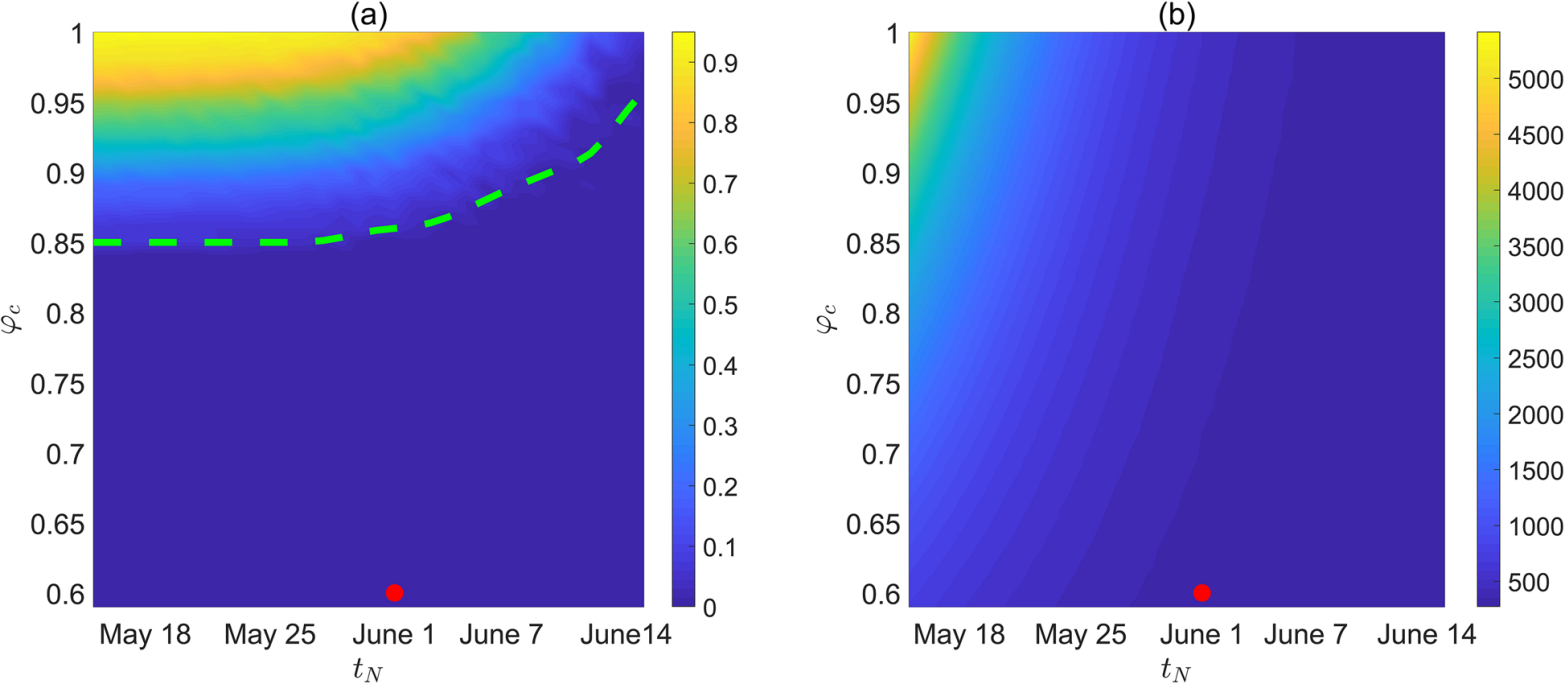
Contour plots of (**a**) the resurgence risk $P\left({\phi }_{c},{t}_{N}\right)$ in June 2022, (**b**) the cumulative number of daily reported cases in June 2022, with respect to the resumed social activity level ratio ${\phi }_{c}$ and the swift relaxation timing ${t}_{N}$. The green dotted curve in (**a**) below which the resurgence risk $P({\phi }_{c},{t}_{N})$ was always 0. Taking strategy 4 as a case, the red points represented the location of (${\phi }_{c},{t}_{N}$), namely, ${\phi }_{c}=0.6$, ${t}_{N}$ was 1 June

Furthermore, by focusing on a relaxation strategy similar to the real case in Shanghai, namely, releasing gradually first after 28 April and then jumping to a constant level at time ${t}_{N}$, we produced contour plots of the resurgence risk $P({\phi }_{c},{t}_{N})$ and the total number of reported cases ${I}_{sum}({\phi }_{c},{t}_{N})$ in June with respect to the resumed social activity level ratio ${\phi }_{c}$ and the swift relaxation timing ${t}_{N}$, as shown in Fig. 5. The results showed that a greater ${\phi }_{c}$ should be combined with a later ${t}_{N}$ to reduce the resurgence risk or maintain a lower total number of reported cases in June. That is, to resume social activity levels close to normal, the swift relaxation timing must be postponed in order to avoid resurgence. For example, if the social activity level was supposed to be resumed to the normal condition (${\phi }_{c}=1$), then the swift relaxation timing should not be earlier than 15 June, otherwise resurgence would probably occur, verifying the case in strategy 6. Furthermore, there was a critical curve of ${\phi }_{c}$ and ${t}_{N}$ (the green dotted curve in Fig. 5(a)) below which the resurgence risk $P({\phi }_{c},{t}_{N})$ was always 0. We observed that the critical curve was close to a horizontal line when ${\phi }_{c}$ was approximately 0.85 and ${t}_{N}$ was between 18 May and 1 June. This also indicated that the resurgence risk $P({\phi }_{c},{t}_{N})$ was more sensitive to the resumed social activity level ratio ${\phi }_{c}$. However, it follows from Fig. 5(b) that the situation seems to be the opposite for the total number of reported cases ${I}_{sum}({\phi }_{c},{t}_{N})$, which is more sensitive to the swift relaxation timing ${t}_{N}$. This was mainly attributed to the earlier relaxation timing being correlated with a larger susceptible population, which may cause more infections once the NPIs were swiftly released. We also observed that given the preset value of ${\phi }_{c}$, postponing ${t}_{N}$ between 18 May and 1 June would significantly reduce the cumulative number of reported cases in June while had little impact on the resurgence risk. That is, later unlocking of the city would not reduce the resurgence risk. This illustrated that a declining trend of the daily number of reported cases should not be taken as the only index to inform the unlocking timing.

**Table 2:** Key parameters of 16 strategies, resurgence probability and the predicted cumulative number of cases under the corresponding strategies

Strategy	${\varvec{\phi }}_{\varvec{k}}$	${\varvec{t}}_{\varvec{N}}$	${\varvec{\phi }}_{\varvec{k}}^{1}\varvec{,}\;{\varvec{\phi }}_{\varvec{k}}^{2}$	${\varvec{t}}_{\varvec{N}}^{1}\varvec{,}\;{\varvec{t}}_{\varvec{N}}^{2}$	${\varvec{\phi }}_{\varvec{c}}$	$\varvec{P}$	${\varvec{I}}_{\varvec{s}\varvec{u}\varvec{m}}$	95%CI
1	1	29 April	--	--	0.26	0%	98	[92–104]
2	1	16 May	--	--	0.34	0%	206	[193–218]
3	1	1 June	--	--	0.41	0%	257	[242–272]
4	1	1 June	--	--	0.6	0%	306	[287–324]
5	1	1 June	--	--	0.8	0%	386	[363–410]
6	1	1 June	--	--	1	53%	514	[481–547]
7	--	--	1, 2.85	1 June, 14 June	0.6	0%	277	[261–294]
8	--	--	1, 5.88	1 June, 14 June	0.8	0%	308	[289–326]
9	--	--	1, 8.92	1 June, 14 June	1	18%	350	[328–371]
10	--	--	1, 1.42	1 June, 28 June	0.6	0%	267	[251–282]
11	--	--	1, 2.94	1 June, 28 June	0.8	0%	280	[263–297]
12	--	--	1, 4.46	1 June, 28 June	1	0%	296	[278–314]
13	2	1 June	--	--	0.57	0%	648	[608–688]
14	3	1 June	--	--	0.72	0%	1604	[1501–1707]
15	4	1 June	--	--	0.88	1%	3928	[3661–4194]
16	5	31 May	--	--	1.01	91%	9607	[8910–10,304]

### Feasibility of regional linked release of NPIs

In the above analysis, the feasibility of various relaxation strategies was investigated by taking Shanghai city as a whole. However, the baseline estimation results showed that the 16 districts in Shanghai could be further divided into two groups according to the time when the number of newly reported cases dropped to 5, the geographical location and the population density, as shown in Table S5. In the 6 districts whose population density was less than 2000 persons/km^2^ and located on the boundary of Shanghai city (including Jiading, Songjiang, Jinshan, Fengxian, Qingpu, Chongming), the number of newly reported cases had dropped to less than 5 before 24 May (17 May on average). We named these 6 districts the boundary-region group (all of them are located on the boundary of the city). For the other 10 districts, whose population density was relatively higher (more than 2000 persons/km^2^) and located in the centre of the city, the newly reported cases had dropped to less than 5 after 25 May. We named theses 10 districts the centre-region group. Then it is reasonable to discuss whether it is feasible to resume social activity between/in districts in the boundary-region group in advance [40, 43, 44].

**Fig. 6:**
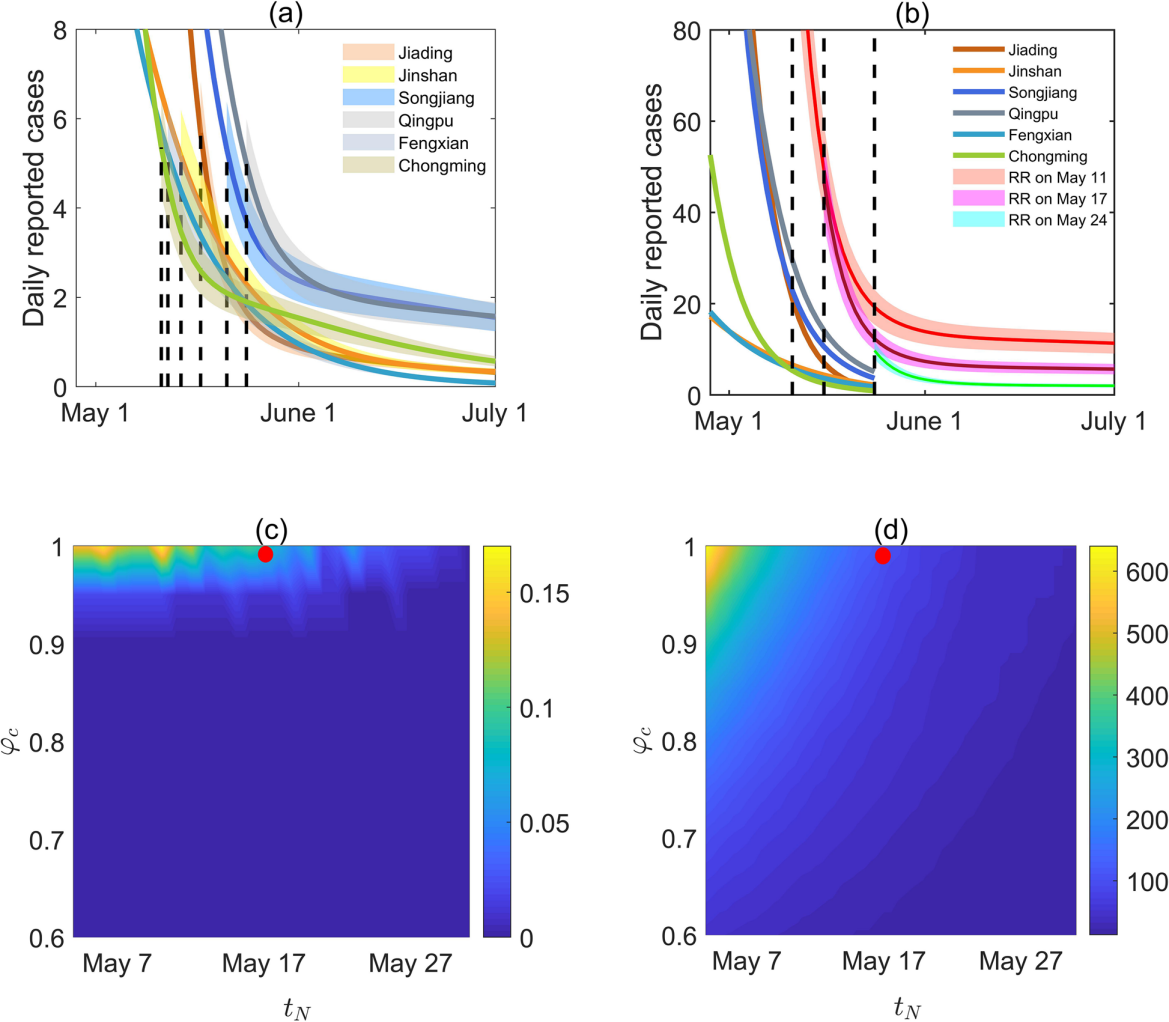
**a** Different situations of relaxation with ${\phi }_{c}=1$ in each district in the boundary-region group when the number of daily reported cases is maintained below 5, **b** the boundary-region group relaxations with ${\phi }_{c}=1$ as a whole by 11, 17 and 24 May, respectively, after the regional linked relaxation, a 10% disturbance was added to the epidemic curve as a shadow to distinguish it. The red points indicate the situation of regional linked relaxations (RR) on 17 May. Contour plots of **c** the resurgence risk $P\left({\phi }_{c},{t}_{N}\right)$ in June 2022, **d** the cumulative number of daily reported cases in June 2022, with respect to the resumed social activity level ratio ${\phi }_{c}$ and the swift relaxation timing ${t}_{N}$. Taking one of the linked relaxation strategies as a case, the red points represented the location of (${\phi }_{c},{t}_{N}$), namely, ${\phi }_{c}=1$, ${t}_{N}$ was 17 May

Based on the baseline estimation in each district, we assumed that NPIs were completely relaxed once the number of daily reported cases in the corresponding district dropped below 5, that is, the social activity level was resumed to the normal (${\phi }_{c}=1$) in each district successively, as shown in Fig. 6(a). The results showed that a resurgence would not occur and the total number of cumulative reported cases in June ${I}_{sum}$ for the boundary-region group was 187 (95%CI: [176–199]), which was 150 cases more than that in the situation without relaxing the NPIs (37 cases in total). Furthermore, we considered that the linked relaxation strategy of resuming social activity in the boundary-region group to a normal level on 11, 17 and 24 May, respectively, which were the earliest, average, latest times for the successive relaxation strategy, as shown in Fig. 6(b). The results revealed that, the total number of reported cases in June adopting the linked relaxation strategy on 11 and 24 May would be 355 (95%CI: [330–379]) and 66 (95%CI: [62–70]), respectively. However, if the boundary-region group adopted the strategy of linked relaxation of NPIs completely (${\phi }_{c}=1$) on 17 May, there would be only a 6% chance of resurgence, and the total number of cases reported in June ${I}_{sum}$ would be 156 (95%CI: [146–167]), close to the successive relaxation strategy. This indicated that Shanghai could safely relax NPIs to resume normal social activity levels in the boundary-region group by mid-May. In addition, the contour plots of the resurgence risk $P({\phi }_{c},{t}_{N})$ (Fig. 6(c)) and the total number of reported cases ${I}_{sum}$ (Fig. 6(d)) in the boundary-region group in June with respect to the resumed social activity level ratio ${\phi }_{c}$ and the regional linkage release timing ${t}_{N}$, showed a similar conclusion as that in the city-wide relaxation strategy (Fig. 5). The relaxation timing of the boundary-region group could be brought forward two days if the social activity levels were resumed to 90% of the normal level, to avoid a resurgence and maintain a low number of reported cases.

In both the regional linked and the city-wide relaxation strategies, we aimed to design effective strategies to relax NPIs as much and early as possible, to avoid a resurgence. To prepare for a long-term fight against the COVID pandemic, a shifting policy aiming to maintain the epidemic below a certain level may be preferred rather than the zero-COVID policy accounting for the continuous mutations of SARS-CoV-2 variants and frequent local outbreaks caused by imported cases. This will be analyzed in depth in the next section.

### Results of optimal control in flattening the epidemic curve

**Fig. 7:**
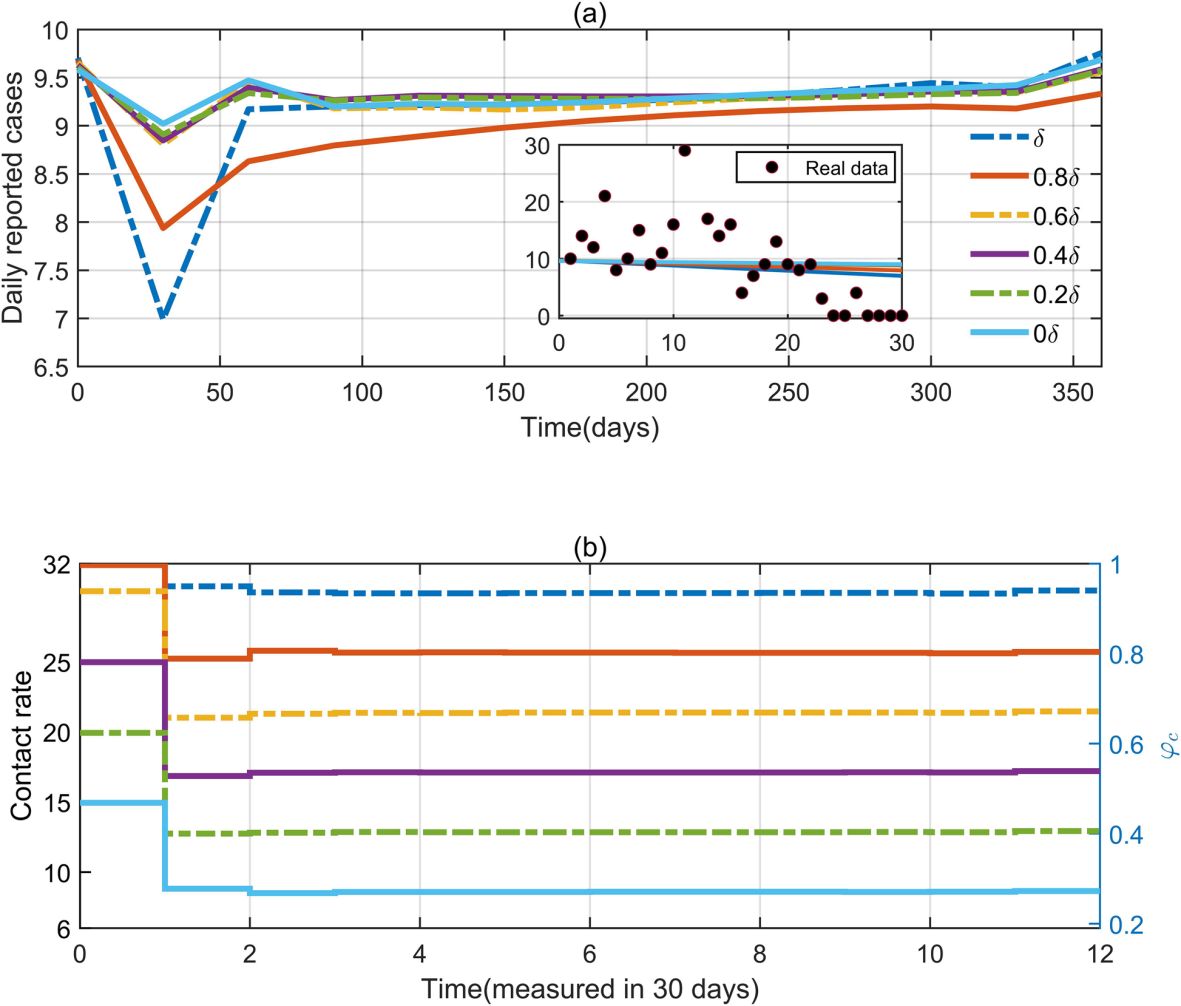
**a** Curves of daily reported numbers of cases optimized with a threshold of 10 cases with different diagnosis rates. The small inset graph shows a comparison between the optimization results of the first 30 days and the real data in Shanghai in June. **b** The optimized contact rate curve

In this section, we explored optimal solutions of the contact rate to maintain the number of daily reported cases below a pre-set threshold within the control duration, under different scenarios with varying testing intensities (different values of the diagnosis rate). Firstly, given a pre-set threshold of 10 of the daily number of reported cases and the control duration of 12 months, we obtained the optimal solutions of the contact rate $c\left(t\right)$ in Fig. 7(b) to maintain the daily number of reported cases below the threshold within the control duration with various diagnosis rates of 0, 0.2$\delta$, 0.4$\delta$, 0.6$\delta$, 0.8$\delta$, $\delta$, where $\delta ={\delta }_{3}$. Here, the initial time for starting the optimal control program was 104 days after 1 March. The corresponding time-varying number of daily reported cases is shown in Fig. 7(a), which decreased at first and then increased, but was maintained below the threshold of 10 throughout the control duration. This phenomenon was mainly due to the activation of optimal control with relaxed NPIs by resuming the contact rate to a relative high level. Then both the optimal contact rate and the number of daily reported cases tended to be constant. Furthermore, a higher diagnosis rate was correlated with a higher optimal contact rate while the number of daily reported cases remained almost unchanged. If the testing strategy was implemented every week (the diagnosis rate $\approx 0.4{\delta }_{3}$), then social activity could only be resumed to about 55% of the normal level, to maintain a low epidemic level. However, if the testing strategy was implemented about every 2.6 days (the diagnosis rate was set as ${\delta }_{3}$), the daily number of reported cases could be maintained below 10 within 12 months even if social activity were to be resumed at 95% of the normal level (${\phi }_{c}=0.95$). This meant that, with a more intensive testing strategy, more social activities could be resumed and people could return to normal life as much as possible but still ensure the epidemic was maintained at a relatively low level, which indicated the necessity of the normalized testing program in helping the resumption of social activity under the optimal control framework. The partial picture showed the comparison between the optimization results of the first 30 days and the real data of Shanghai in June. Obviously, the reported numbers of cases in Shanghai did indeed follow a low-level epidemic pattern in the average sense.

We also present the optimization results when the acceptable threshold was preset as 100 or 1000 (see SI Fig. 2). A similar phenomenon to that of the daily number of reported cases decreasing at first and then increasing was observed during the first two months of the control duration. The results showed that for the case of a threshold of 10 or 100, the optimal contact rate was supposed to be almost ${c}_{1}$ with $\delta ={\delta }_{3}$, while for the case of a threshold of 1000, the optimal contact rate was supposed to be less than 80% ${c}_{1}$ with $\delta ={\delta }_{3}$. This indicated that, if the goal was to maintain the number of daily reported cases below 10 or 100, then the social activity level could be resumed to almost 100% (${\phi }_{c}\approx 95\%$) of the normal level. However, if the goal was to maintain the number of daily reported cases below 1000, the social activity level could only be resumed to 80% of the normal level at most, even with a strict testing strategy.

### Retrospective analysis in the city-based pattern

We observed the obvious feature of the current round of the epidemic in Shanghai is that the government responded late in the early stage, which led to the rapid growth of the index in the early stage, resulting in such a huge scale of the epidemic, compared to other epidemics in China in the past two years. We therefore did some retrospective analysis by assuming that the control interventions were implemented τ days in advance with τ = 0,3,5,7,10,14., as shown in SI Fig. 3. It was found that if all control interventions were brought forward by two weeks, the cumulative numbers of cases are reduced by around 96.4%. We further verified the importance of the diagnosis rate in the retrospective analysis of SI Fig. 4(a)(b). If the diagnosis rate is only $0.5{\delta }_{3}$ from the beginning of the epidemic, then even if the social activity rate ${\phi }_{c}$ has been maintained at the level of 0.7, it will lead to a total of 7.44 million people infected. If the diagnosis rate is maintained at ${\delta }_{3}$ from the beginning, then even without any restrictions on social activities, the infection peak of the Shanghai epidemic will not exceed 25,000. In other words, this will not be worse than the current situation. SI Fig. 4(c) simulates that when NPIs are strict enough with $q={q}_{3}, \delta ={\delta }_{3}$, even if the government responds late within a certain range, it will not cause a huge outbreak of the epidemic which will be out of control. Our discussion of switching time points for tightening or loosening control interventions is presented in SI Fig. 4(d), where it is shown that when both the diagnosis rate $\delta ={\delta }_{3}$ and the isolation rate $q={q}_{3}$ are at their highest levels, the control of the social activity rate can be adjusted according to the number of daily reported cases being dynamically adjusted to control the epidemic at a certain low level.

## Discussion

The aggressive zero-COVID policy was shown to work remarkably well before the Omicron variants swept through mainland China [4, 9, 45, 46]. However, the high transmissibility of SARS-CoV-2 variants is persistently increasing the social and economic costs of control, even challenging the affordability of the control and prevention measures [47, 48]. This drives researchers to explore the possibility of relaxing the control interventions as much and as early as possible while keeping the epidemic under control, i.e., avoiding big upsurges of infected cases. Correspondingly, in this study, we considered two different controlling targets. One was to still control the cases down to zero with no resurgence, but choosing to partially and gradually relax the interventions at the cost of prolonging the epidemic duration and slightly increasing the cumulative number of infected cases. The other was to maintain the epidemic at a low level rather than controlling the cases down to zero.

A mathematical model was proposed to investigate the feasibility of various relaxation strategies of the outbreak caused by the SARS-CoV-2 Omicron variant in Shanghai city focusing on the two different control targets. We firstly calibrated the model by employing multi-source data (Fig. 3) and estimated the parameters of the epidemic in Shanghai city and in each district (Tables 1 and S1-S4), respectively. A long tail of the epidemic in Shanghai was observed and the 16 districts were divided into two groups according to the baseline estimation and prediction results. Our fitting results on the data can be mutually verified with two studies [40, 49], including the simulation results based on deep neural networks. Then to avoid a big resurgence, we considered a step-by-step relaxation strategy with two patterns: the city-based and the regional linked (district-based) relaxation strategies after 28 April. For the city-based relaxation strategy, we initially designed 16 strategies centred on gradual relaxation of control interventions in two or three stages. We observed that there were strategies allowing more social activity or later relaxation timings while ensuring zero resurgence risk at the cost of a slightly increased number of reported cases (strategies 4, 5, 7, 8, 10, 11, 12 shown in Fig. 4; Table 2) compared with the baseline situation. We found that strategy 4, in which NPIs were swiftly released on 1 June to resume the social activity level to 0.6 times of the normal level, was the closest relaxation strategy to that adopted by Shanghai. The contour plots in Fig. 5 revealed that the social activity level could be further resumed to higher social activity levels without causing resurgence or causing more reported cases. For the district-based release strategy, the 16 districts were divided into boundary-region and centre-region groups. We found that the social activity in 6 of the 16 districts (the boundary-region group) could be completely resumed (to 100% of the normal level) 14 days in advance compared with the city-based relaxation strategy. Even if people were allowed to flow freely between different districts in the boundary-region group, no resurgence occurred. The results reflected that there was supposed to be a more adventurous relaxation strategy by resuming more social activity in Shanghai. A regional linked release strategy of unleashing several districts (the boundary-region group) in advance was recommended and more attention should be paid to controlling the epidemic in the centre-region group, which is in accordance with the results given by a retrospective statistical investigation [40, 43, 44]. With a purpose to control the epidemic at a low level, we also searched for optimal solutions of the contact rate using optimal control theory. Given a pre-set threshold of 10 of the daily numbers of reported cases and a control duration of 12 months, we obtained the optimal solutions of the contact rate $c\left(t\right)$ to maintain the daily number of reported cases below the threshold within the control duration with various diagnosis rates. We found that a higher diagnosis rate correlated with a higher optimal contact rate while the number of daily reported cases remained almost unchanged. This means that more social activities can be resumed through more intensive detection strategies, so that people can resume their normal lives as much as possible, while ensuring that the epidemic remains at a low level. This showed that it is necessary for the normalized testing program / regular nucleic acid testing to be maintained in order to help to restore social activities under the optimal control framework [50–52]. However, this was different from the case where the threshold was 10 when the threshold is too large, say 1000, then even if we keep the maximum detection rate, the social activity rate will not recover to 80%. Thus, only when the number of daily reported cases is controlled to a very small number and then through optimization measures to transition the zero-COVID policy to a low-level epidemic can we obtain the expected effect, otherwise social activities will still be significantly restricted. It is also worth noting that the various levels of contact rates given in this paper are only the simulation results at the mathematical level, and policy makers may refer to the contact rates ${c}_{1}$ to ${c}_{4}$ of different periods in the model fitting part as a reference.

From an overall perspective, we found that the exponential growth in the early stage of the epidemic in Shanghai led to such a large scale epidemic, which is closely related to the late response of the government in the early stage, and thus reflects a new feature that has not been seen in other outbreaks in China in the past two years. Faced with this dilemma, Shanghai’s lockdown was necessary [53]. The retrospective analysis shows that the cumulative number of cases in this outbreak could have been reduced by about 96.4% if all control measures had been implemented two weeks in advance as a whole. Lou et al. [54] reached similar conclusions in their retrospective analysis of the Shanghai outbreak. If the testing strategy was implemented about every 2.6 days, the peak number of reported infections per day will not exceed 25,000, even if there are no restrictions on social activities. These results highlight the effectiveness and importance of early nucleic acid testing [51]. In addition, it has also shown that as long as NPIs are strong enough, that is, the diagnostic rate $\delta$ and isolation rate $q$ are both high, then the government’s response time is much less sensitive to outbreaks. This indicates that strong control efforts have quite significant inhibitory effects on the spread of COVID-19 [55]. Under the same conditions, social activity control can be dynamically adjusted according to the daily number of reported cases to keep the outbreak at a low level. A retrospective analysis of the diagnosis rate confirms its importance.

It is worth noting that our step-by-step relaxation strategies are designed based on linear functions, which is an idealized situation. It is well known that the effect of policy implementation is not direct reality, and any policy has a certain lag. In reality, the relaxation of control measures is likely to be reflected by nonlinear or even non-monotonic change functions. For Eqs. (3) and (4) with simple forms, the selection of more complex and exquisite functions may need further attempts. In addition, the optimization problem considered in this paper does not take into account economic benefits, while the cost of nucleic acid screening in practical scenarios and other factors should not be ignored by policy makers. The optimal results given by the model may still be a short distance from the real implementation, which is of course a crucial issue in the connection between mathematics and real life. In particular, the time-varying control strength (i.e., contact rate) which is measured in 30 days, as shown in Fig. 7(b), still appears insufficient for the precision of the control. As for the control of dynamically adjusted social activities in the retrospective analysis, the scenario we considered still appears to be slightly limited, and for example, SI Fig. 4(d) shows only a tiny fraction of the exploration of dynamic optimization. A more flexible approach to timing and thresholds would help, but it is also a challenge. It should not be overlooked that all of the above suggest some needs and directions for future research.

## Conclusions

Appropriate release strategies could allow earlier and more resumption of social activity without causing resurgence in the local outbreak caused by the SARS-CoV-2 Omicron variant in Shanghai. Particularly, a regional linked release strategy was recommended to completely relax the boundary-region group earlier and more attention should be paid to the centre-region group. The results of optimal control provide a viable case for the transformation of the COVID-19 epidemic from pandemic to endemic. It is necessary to maintain the normalized testing program in helping the resumption of social activity. With a more intensive testing strategy, more social activities could be resumed and people could return to normal life as much as possible but still ensure that the epidemic was maintained at a relatively low level.

## Supplementary Information

**Additional file 1.**

## Data Availability

The data used for this study are published by the National Health Commission of the People’s Republic of China, publicly available at the following link: http://en.nhc.gov.cn/. The dataset has also been uploaded to GitHub for ease of search and is publicly available at the following link: https://github.com/SNWZXS101/Data-of-Shanghai-COVID-19.
