# Supplementary material for: Lessons drawn from Shanghai for controlling highly transmissible SARS-CoV-2 variants: insights from a modelling study
Source: BMC Infect Dis. 2023 May 16;23:331. doi: 10.1186/s12879-023-08316-7 (PMC10186324; doi:10.1186/s12879-023-08316-7)
Supplement: Supplementary file 1 — Additional file 1. [file 12879_2023_8316_MOESM1_ESM.docx]

**Supplementary Material: Lessons drawn from Shanghai for controlling the highly transmissible SARS-CoV-2 variants：insights from a modelling study**

**Hao Wang^1^, Tangjuan Li^2^, Huan Gao^1^, Chenxi Huang^1^, Biao Tang^2^, Sanyi Tang^1^, Robert A. Cheke^3^, Weike Zhou^4*^**

**^1^** School of Mathematics and Statistics, Shaanxi Normal University, Xi’an 710062, PR China

**^2^** School of Mathematics and Statistics, Xi’an Jiaotong University, Xi’an 710049, PR China

^3^ Natural Resources Institute, University of Greenwich at Medway, Central Avenue, Chatham Maritime, Kent, ME4 4TB, UK

^4^  School of Mathematics, Northwest University, Xi’an 710127, PR China

*** Correspondence:** [wkzhou@snnu.edu.cn](mailto:wkzhou@snnu.edu.cn); zhouweikexjtu@163.com

**Model-free estimation method of the effective reproduction number**

To evaluate the transmission ability of the Omicron variant spread in Shanghai and get information on the effectiveness of control interventions during the outbreak, we estimated the effective reproduction number $R_{t}$ by using the renewal equation method. The renewal equation method seems to be a widely used method at present as a model-free method [1-5]. Note that there are others model-free methods to calculate the effective reproduction number, such as the EpiNow2 method [6] and EpiEstim method [7]. However, both of them need high-quality data sources such as incubation period distribution and even delay of case reports in addition to new case data. The renewal equation method requires only daily new cases and estimates of intergenerational intervals, both of which are readily available. Let $M_{t}$ be the number of newly reported cases on day $t$ and assume that $M_{t}$ follows a Poisson distribution with mean $\lambda_{t}$, namely, $M_{t}|M_{0},\ldots,M_{t-1}\sim\mathrm{Poisson}(\lambda_{t})$. Then by using $\lambda_{t}=R_{t}\sum_{\tau=1}^{t} g_{\tau}I_{t-\tau}$ within the Bayesian framework, where $g_{\tau}$ is the discretized generation interval, which is assumed to be a Gamma distribution, an analytical expression of the posterior distribution of $R_{t}$ could be obtained by assuming the prior distribution of $R_{t}$ is a Gamma distribution.

In detail, the shape and scale parameters for the prior Gamma distribution of $R_{t}$ are assumed to be 1 and 5, respectively, and the mean and standard derivation of the generation interval are 2.35 and 2.95, respectively. The effective reproduction number can be calculated by setting it to be the median of the posterior distribution and the 0.025 and 0.975 quantiles were used to calculate the 95% confidence intervals.

Based on the above method and using the epidemic data of the outbreak in Shanghai between 1 March and 31 May, we obtained the estimation of the effective reproduction number, as shown in SI Fig. 3. Note that, we estimated the effective reproduction number at 1 May as 0.74, which is in line with the estimation from Shanghai New Crown Epidemic Prevention and Control Leading Group Office [8].

**Timeline of the implementation of interventions in Shanghai**

- 28 March: Closed-off management and population-based nucleic acid testing were carried out by districts;
- 2 April: Citywide lockdown was implemented in Shanghai;
- 11 April: Three control modes were strictly implemented by areas;
- 22 April: Partition of areas implementing three different control modes were adjusted;
- 28 April: Dynamic adjustment for partition of areas implementing three different control modes began.
- 16 May: The phased resumption of work and production started.
- 1 June: The normal production and living order of the city would be fully restored.

**Optimal control problem**

To control the epidemic below a preset level, we can transfer it to an optimal control problem, given

(a)

(b)

(c)

(d)

(e)

(f)

$$\min_{u} \int_{0}^{T} {(1-u(t))}^{2}dt$$

*s.t.*

equations (1)

$$0\leq u(t)\leq1$$

$$N(\delta I+\delta_{q}I_{q})\leq N_{max}$$

$$c(t)=c(k\Delta t),t\in[k\Delta t,(k+1)\Delta t],k=0,1,...,K$$

$\Delta t=1$ week,

where (a) is the objective function, namely, the normalized economic cost of social distancing. (b) are ordinary differential equations integrating prevention and control measures and describing the epidemic trend of COVID-19, where $c\left( t \right)=c_{min}+\left( c_{max}-c_{min} \right)u\left( t \right)$ represents the average number of contacts per infected person in the risk area at time $t$; (c) represents constraints on normalized prevention and control measures; (d) indicates that the number of new reported cases does not exceed some critical value; (e) and (f) mean that countermeasures are kept constant over one week, i.e. $\Delta t$ is one week, $K$ is the total number of weeks considered, *T* is the total number of days considered and $N$ is the total number of original people. Here, the equation (1) is expressed as:

$$\begin{aligned} &\frac{dS}{dt}=\lambda S_{q}-c(t)\beta\frac{SI}{S+E+I\text{+}R}-c(t)\left( 1-\beta\right)q\frac{SI}{S+E+I+R} \\ &\frac{dE}{dt}=c(t)\beta\left( 1-q \right)\frac{SI}{S+E+I+R}-\sigma E \\ &\frac{dI}{dt}=\sigma E-\gamma I-\delta I \\ &\frac{dS_{q}}{dt}=c(t)\left( 1-\beta\right)q\frac{SI}{S+E+I+R}-\beta_{q}\frac{S_{q}I_{q}}{S_{q}+E_{q}+I_{q}}-\lambda S_{q} \\ &\frac{dE_{q}}{dt}=c(t)\beta q\frac{SI}{S+E+I+R}+\beta_{q}\frac{S_{q}I_{q}}{S_{q}+E_{q}+I_{q}}-\sigma E_{q} \\ &\frac{dI_{q}}{dt}=\sigma E_{q}-\delta_{q}I_{q} \\ &\frac{dH}{dt}=\delta I+\delta_{q}I_{q}-dH-\gamma_{H}H \\ &\frac{dR}{dt}=\gamma I+\gamma_{H}H \\ &\frac{dD}{dt}=dH \end{aligned}(1)$$

where, $c\left( t \right)=c_{min}+\left( c_{max}-c_{min} \right)u\left( t \right)$，$u\left( t \right)\in[0,1]$, $c_{max}=c_{1}$，$c_{min}=c_{3}$.

**
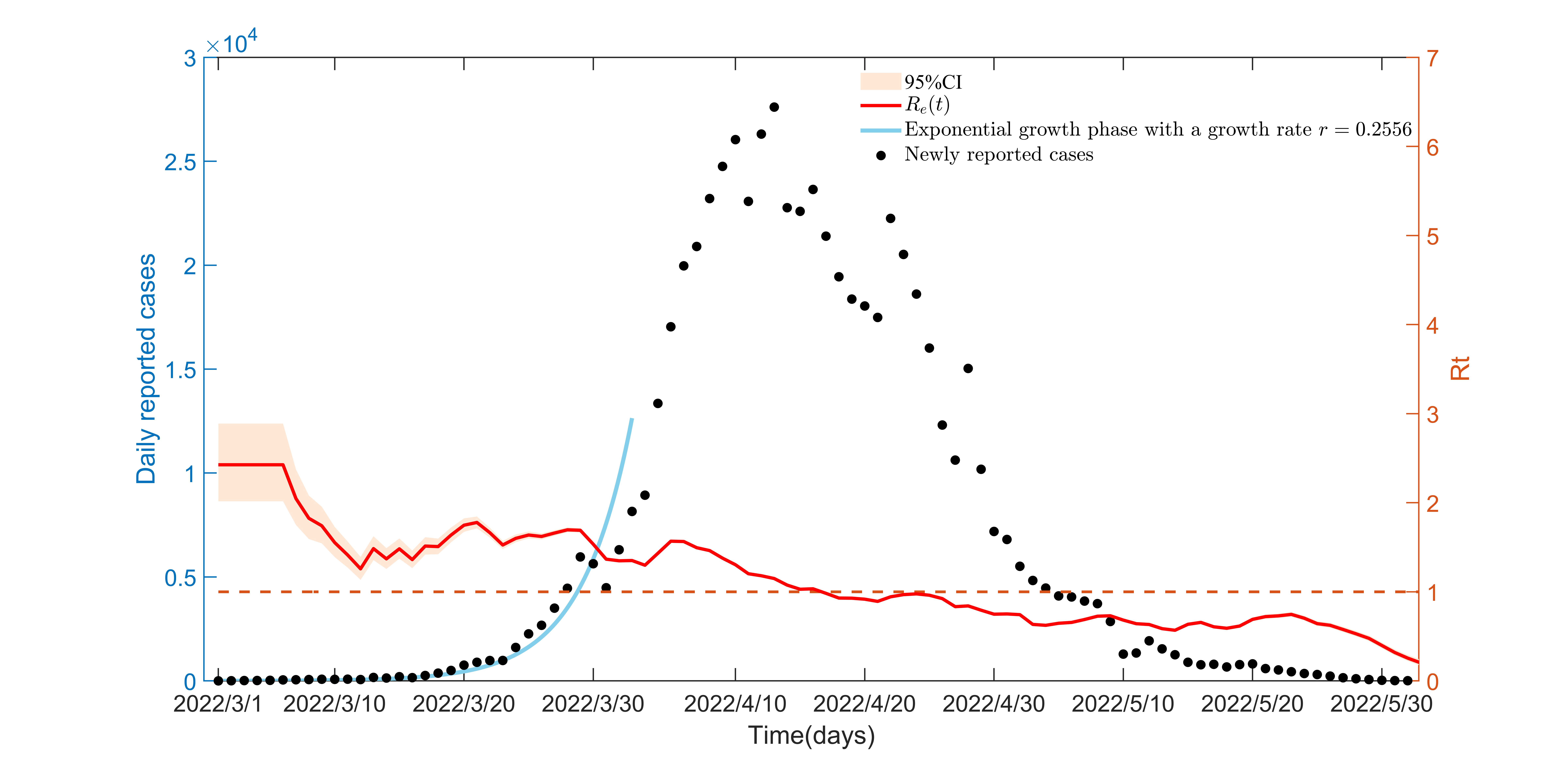
SI Figures**

**SI Fig. 1** Effective reproduction number of the local outbreak of Shanghai using the model free estimation method. The solid red line represents the mean of reproduction numbers, while the shaded area represents the 95% confidence interval. Black dots are real confirmed case data and the dotted line is the threshold 1 for the reproduction number. The trend of case growth in the first 33 days was fitted with an exponential growth curve and represented by a lavender line.

**
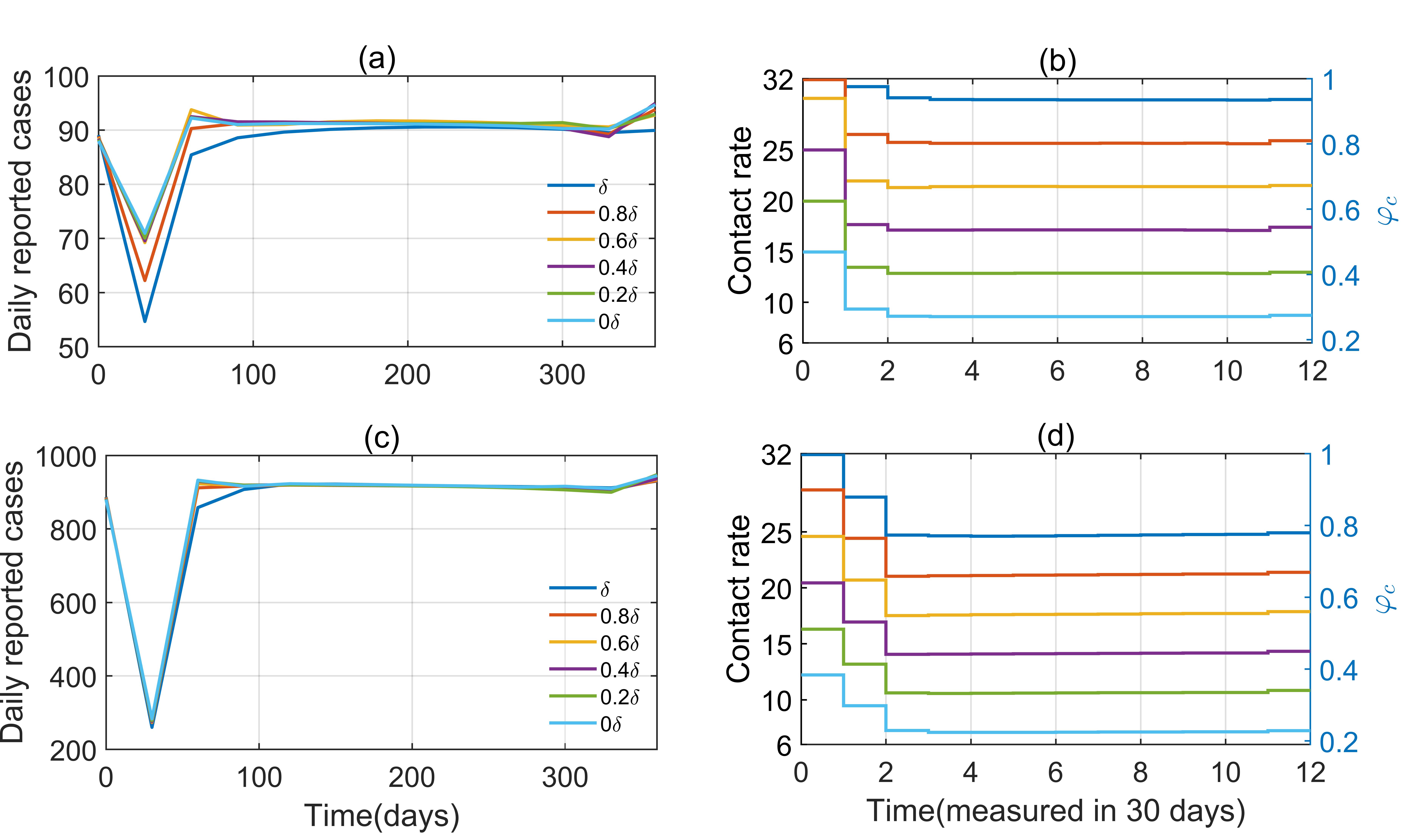
**

**SI Fig. 2** (a) Curves of daily reported numbers of cases optimized with a threshold of 100 cases under different $\delta$ levels, and (b) the corresponding optimized contact rate. (c) Curves of daily numbers of reported cases optimized with a threshold of 1000 cases under different $\delta$ levels, and (d) the corresponding optimized contact rate.



**SI Fig. 3** Retrospective analysis by assuming that the control interventions were implemented $\tau$ days in advance with $\tau=0,3,5,7,10,14$. Correspondingly, the values of control related parameters are assumed to shift forward for $\tau$ days with $c\left( t \right)=c\left( t+\tau\right), \delta\left( t \right)=\delta\left( t+\tau\right),q=q(t+\tau)$. Here, FR means free (non-quarantined) region and QR denotes the quarantine region. We also listed the peak time, end time and the final epidemic size in Table S7. The curve of the entire epidemic process in Shanghai was simulated under six different T-values: (a) daily new cases in FR, (b) daily new cases in QR, (c) cumulative cases in FR, and (d) cumulative cases in QR. The results show that the final epidemic size can be reduced by half if we take the enhanced action 3 days in advance while the cumulative numbers of cases are reduced by around 96.4% when $\tau=14$. However, we find that there 85 days still be needed to the end time for zero-COVID even by implementing the interventions 14 days in advance.


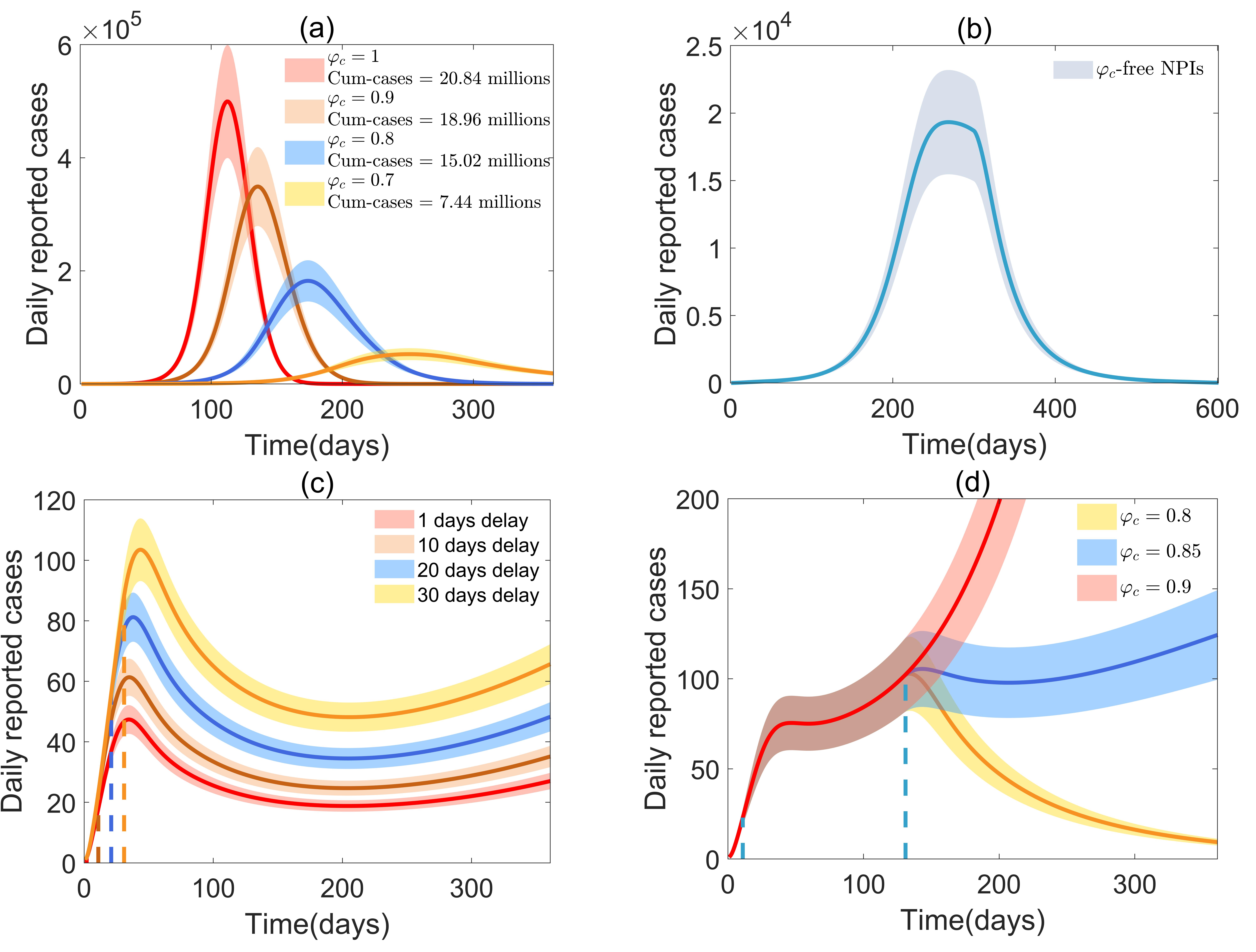


**SI Fig. 4** (a) After the diagnosis rate is reduced to 0.5 times ($\delta=0.5\delta_{3}$), the overall trend of the epidemic curve and the final size of the epidemic with different $\varphi_{c}$，(b) The trend of the epidemic with $\varphi_{c}$-free NPIs, that is to maintain the highest level of tracing and isolation and nucleic acid testing($q=q_{3}, \delta=\delta_{3}$. The same condition applies to (c) and (d) ), but completely relax social distancing control, (c) Effects of NPIs with $\varphi_{c}=0.85$ on delayed response of different days, (d) Tighten NPIs to $\varphi_{c}=0.9$ on day 10, followed by adjustments to $\varphi_{c}=0.8, 0.85, 0.9$ on day 130 when the epidemic re-emerges. In addition, all shaded areas represent a 15% random disturbance to the simulation results

**SI Tables**

**Table S1** Values of parameters and variables for districts 1 to 4

| **Parameters** |  | **Value(95%CI)** | | | |
| --- | --- | --- | --- | --- | --- |
|  |  | **Pudong New Area** | **Huangpu** | **Xuhui** | **Changning** |
| $c$ | $c_{1}$  $c_{2}$  $c_{3}$  $c_{4}$  $k$ | 31.600(31.034-32.165)  16.292(15.683-16.900)  8.301(7.808-8.795)  10.256(8.829-11.682)  0.283(0.247-0.319) | 27.322(26.593-28.051)  14.731(14.439-15.024)  7.835(7.617-8.053)  9.501(9.121-9.881)  0.312(0.296-0.329) | 24.252(22.245-26.259)  10.781(9.345-12.216)  8.896(6.120-11.673)  10.111(7.686-12.535)  0.277(0.227-0.328) | 23.677(22.864-24.489)  14.141(13.699-14.584)  8.419(7.919-8.919)  8.695(7.937-9.453)  0.2539(0.2368-0.2710) |
| $\beta$ |  | 0.082(0.081-0.083) | 0.077(0.076-0.078) | 0.101(0.087-0.115) | 0.084(0.082-0.086) |
| $\beta_{q}$ |  | 0.833(0.754-0.906) | 0.603(0.588-0.617) | 0.681(0.450-0.966) | 0.914(0.859-0.970) |
| $\lambda$ |  | 1/14 | 1/14 | 1/14 | 1/14 |
| $\sigma$ |  | 1/3 | 1/3 | 1/3 | 1/3 |
| $q$ | $q_{1}$  $q_{2}$  $q_{3}$ | 0.592(0.577-0.606)  0.707(0.696-0.718)  0.791(0.779-0.803) | 0.619(0.612-0.627)  0.757(0.750-0.764)  0.900(0.893-0.907) | 0.567(0.515-0.619)  0.717(0.653-0.782)  0.809(0.727-0.890) | 0.601(0.586-0.616)  0.657(0.647-0.667)  0.819(0.804-0.835) |
| $\delta$ | $\delta_{1}$  $\delta_{2}$  $\delta_{3}$ | 0.220(0.208-0.233)  0.320(0.301-0.340)  0.438(0.412-0.465) | 0.145(0.139-0.150)  0.224(0.208-0.240)  0.307(0.284-0.329) | 0.239(0.179-0.299)  0.304(0.249-0.360)  0.416(0.341-0.467) | 0.220(0.207-0.233)  0.244(0.230-0.257)  0.334(0.315-0.352) |
| $\delta_{q}$ |  | 0.631(0.597-0.665) | 0.415(0.403-0.428) | 0.602(0.454-0.750) | 0.496(0.466-0.526) |
| $\gamma$ |  | 1/7 | 1/7 | 1/7 | 1/7 |
| $\gamma_{H}$ |  | 1/7 | 1/7 | 1/7 | 1/7 |
| $d$ |  | 4.736 (4.311-5.160)*e-5 | 3.885 (3.543-4.227)*e-5 | 4.811 (3.693-0.593)*e-5 | 4.519 (4.255-4.784)*e-5 |
| **Initial value** |  | **Value(95%CI)** | | | |
|  |  | **Pudong New Area** | **Huangpu** | **Xuhui** | **Changning** |
| $S(0)$ |  | 5681500 | 662000 | 1113100 | 693100 |
| $E(0)$ |  | 13.059(11.676-14.443) | 9.102(7.944-10.261) | 5.110(0.761-9.458) | 7.522(6.226-8.817) |
| $I(0)$ |  | 1 | 0 | 0 | 1 |
| $S_{q}(0)$ |  | 0 | 0 | 0 | 0 |
| $E_{q}(0)$ |  | 0 | 0 | 0 | 0 |
| $I_{q}(0)$ |  | 2 | 1 | 4 | 1 |
| $H(0)$ |  | 0 | 0 | 0 | 0 |
| $R(0)$ |  | 0 | 0 | 0 | 0 |
| $D(0)$ |  | 0 | 0 | 0 | 0 |

**Table S2** Value of parameters and variables for districts 5 to 8

| **Parameters** |  | **Value(95%CI)** | | | |
| --- | --- | --- | --- | --- | --- |
|  |  | **Jing’an** | **Putuo** | **Hongkou** | **Yangpu** |
| $c$ | $c_{1}$  $c_{2}$  $c_{3}$  $c_{4}$  $k$ | 27.878(26.610-29.147)  14.711(14.474-14.947)  5.787(5.630-5.944)  8.534(8.214-8.855)  0.560(0.540-0.579) | 24.157(23.485-24.829)  12.781(12.126-13.435)  3.536(3.214-3.858)  4.690(4.320-5.059)  0.279(0.260-0.297) | 24.898(23.939-25.858)  14.391(14.019-14.762)  5.736(5.189-6.283)  9.142(8.681-9.602)  0.182(0.155-0.209) | 31.010(29.983-32.036)  19.016(18.322-19.710)  7.553(7.363-7.742)  9.234(8.771-9.697)  0.274(0.255-0.294) |
| $\beta$ |  | 0.092(0.090-0.094) | 0.091(0.088-0.093) | 0.090(0.088-0.093) | 0.088(0.087-0.089) |
| $\beta_{q}$ |  | 0.488(0.443-0.530) | 0.336(0.308-0.365) | 0.552(0.518-0.587) | 0.511(0.472-0.547) |
| $\lambda$ |  | 1/14 | 1/14 | 1/14 | 1/14 |
| $\sigma$ |  | 1/3 | 1/3 | 1/3 | 1/3 |
| $q$ | $q_{1}$  $q_{2}$  $q_{3}$ | 0.661(0.649-0.673)  0.778(0.773-0.782)  0.881(0.870-0.891) | 0.675(0.671-0.680)  0.576(0.748-0.765)  0.906(0.882-0.929) | 0.665(0.652-0.679)  0.744(0.730-0.758)  0.872(0.850-0.893) | 0.719(0.713-0.725)  0.806(0.802-0.811)  0.964(0.956-0.968) |
| $\delta$ | $\delta_{1}$  $\delta_{2}$  $\delta_{3}$ | 0.158(0.142-0.174)  0.226(0.211-0.240)  0.309(0.302-0.329) | 0.156(0.150-0.162)  0.239(0.220-0.257)  0.327(0.301-0.352) | 0.142(0.133-0.150)  0.168(0.159-0.177)  0.230(0.218-0.242) | 0.175(0.165-0.185)  0.238(0.220-0.255)  0.326(0.301-0.349) |
| $\delta_{q}$ |  | 0.372(0.342-0.401) | 0.355(0.344-0.367) | 0.443(0.424-0.461) | 0.329(0.318-0.340) |
| $\gamma$ |  | 1/7 | 1/7 | 1/7 | 1/7 |
| $\gamma_{H}$ |  | 1/7 | 1/7 | 1/7 | 1/7 |
| $d$ |  | 3.477(3.179-3.775)*e-5 | 3.429(3.171-3.686)*e-5 | 3.336(3.063-3.608)*e-5 | 3.345(3.002-3.688)*e-5 |
| **Initial value** |  | **Value(95%CI)** | | | |
|  |  | **Jing’an** | **Putuo** | **Hongkou** | **Yangpu** |
| $S(0)$ |  | 975700 | 1239800 | 757500 | 1242500 |
| $E(0)$ |  | 3.447(3.093-3.802) | 4.185(3.483-4.887) | 7.035(6.137-7.933) | 9.864(8.245-11.481) |
| $I(0)$ |  | 0 | 1 | 0 | 1 |
| $S_{q}(0)$ |  | 0 | 2 | 0 | 6 |
| $E_{q}(0)$ |  | 0 | 0 | 0 | 0 |
| $I_{q}(0)$ |  | 1 | 1 | 1 | 0 |
| $H(0)$ |  | 0 | 0 | 0 | 0 |
| $R(0)$ |  | 0 | 0 | 0 | 0 |
| $D(0)$ |  | 0 | 0 | 0 | 0 |

**Table S3** Value of parameters and variables for districts 9 to 12

| **Parameter** | | **Value(95%CI)** | | | |
| --- | --- | --- | --- | --- | --- |
|  |  | **Minhang** | **Baoshan** | **Jiading** | **Jinshan** |
| $c$ | $c_{1}$  $c_{2}$  $c_{3}$  $c_{4}$  $k$ | 24.136(23.340-24.933)  10.733(10.419-11.045)  5.697(5.477-5.918)  8.600(8.408-8.792)  0.367(0.345-0.389) | 31.217(30.274-32.161)  25.288(23.860-26.715)  21.258(20.335-22.180)  13.166(12.302-14.030)  0.280(0.252-0.308) | 25.440(24.724-26.156)  13.800(13.290-14.310)  6.282(6.022-6.542)  10.056(9.706-10.406)  0.315(0.293-0.337) | 22.996(21.762-24.230)  5.642(5.355-5.930)  5.276(4.896-5.654)  5.246(5.100-5.391)  0.296(0.285-0.307) |
| $\beta$ |  | 0.089(0.088-0.091) | 0.077(0.076-0.078) | 0.099(0.096-0.101) | 0.093(0.01-0.095) |
| $\beta_{q}$ |  | 0.966(0.916-1) | 0.646(0.575-0.721) | 0.532(0.493-0.565) | 0.573(0.526-0.622) |
| $\lambda$ |  | 1/14 | 1/14 | 1/14 | 1/14 |
| $\sigma$ |  | 1/3 | 1/3 | 1/3 | 1/3 |
| $q$ | $q_{1}$  $q_{2}$  $q_{3}$ | 0.602(0.588-0.617)  0.743(0.731-0.755)  0.854(0.845-0.863) | 0.693(0.687-0.699)  0.789(0.783-0.795)  0.922(0.912-0.931) | 0.660(0.648-0.672)  0.858(0.845-0.872)  0.922(0.914-0.930) | 0.677(0.665-0.688)  0.764(0.755-0.773)  0.814(0.808-0.820) |
| $\delta$ | $\delta_{1}$  $\delta_{2}$  $\delta_{3}$ | 0.228(0.215-0.241)  0.241(0.224-0.258)  0.330(0.306-0.353) | 0.201(0.188-0.214)  0.281(0.269-0.292)  0.385(0.368-0.400) | 0.233(0.225-0.241)  0.274(0.264-0.285)  0.375(0.362-0.390) | 0.197(0.185-0.209)  0.241(0.226-0.256)  0.330(0.310-0.351) |
| $\delta_{q}$ |  | 0.455(0.433-0.477) | 0.424(0.389-0.460) | 0.389(0.374-0.404) | 0.689(0.662-0.715) |
| $\gamma$ |  | 1/7 | 1/7 | 1/7 | 1/7 |
| $\gamma_{H}$ |  | 1/7 | 1/7 | 1/7 | 1/7 |
| $d$ |  | 4.330(3.959-4.701)*e-5 | 3.475(3.208-3.741)*e-5 | 4.632(4.411-4.854)*e-5 | 4.512(4.346-4.678)*e-5 |
| **Initial value** |  | **Value(95%CI)** | | | |
|  |  | **Minhang** | **Baoshan** | **Jiading** | **Jinshan** |
| $S(0)$ |  | 2653500 | 2235200 | 1834300 | 822800 |
| $E(0)$ |  | 9.144(7.681-10.607) | 10.456(9.476-11.436) | 6.498(5.936-7.060) | 2.991(2.533-3.448) |
| $I(0)$ |  | 1 | 0 | 0 | 0 |
| $S_{q}(0)$ |  | 2 | 2 | 0 | 0 |
| $E_{q}(0)$ |  | 0 | 0 | 0 | 0 |
| $I_{q}(0)$ |  | 1 | 6 | 6 | 1 |
| $H(0)$ |  | 0 | 0 | 0 | 0 |
| $R(0)$ |  | 0 | 0 | 0 | 0 |
| $D(0)$ |  | 0 | 0 | 0 | 0 |

**Table S4** Value of parameters and variables for districts 13 to 16

| **Parameter** | | **Value (95%CI)** | | | |
| --- | --- | --- | --- | --- | --- |
|  |  | **Songjiang** | **Qingpu** | **Fengxian** | **Chongming** |
| $c$ | $c_{1}$  $c_{2}$  $c_{3}$  $c_{4}$  $k$ | 24.564(24.384-24.744)  14.974(14.955-14.993)  8.976(8.950-9.002)  7.578(7.000-8.155)  0.293(0.276-0.309) | 25.949(25.193-26.705)  10.674(10.318-11.031)  4.579(4.430-4.729)  7.788(7.331-8.244)  0.5764(0.535-0.617) | 24.272(23.945-24.600)  6.462(6.335-6.589)  6.165(6.016-6.313)  7.565(7.420-7.711)  0.668(0.638-0.708) | 26.445(26.160-26.731)  12.410(11.421-13.400)  7.573(7.210-7.935)  9.963(9.947-9.978)  0.365(0.346-0.384) |
| $\beta$ |  | 0.099(0.098-0.099) | 0.097(0.096-0.098) | 0.078(0.077-0.079) | 0.155(0.152-0.157) |
| $\beta_{q}$ |  | 0.683(0.639-0.736) | 0.679(0.638-0.728) | 0.402(0.373-0.437) | 1.017(0.935-1.074) |
| $\lambda$ |  | 1/14 | 1/14 | 1/14 | 1/14 |
| $\sigma$ |  | 1/3 | 1/3 | 1/3 | 1/3 |
| $q$ | $q_{1}$  $q_{2}$  $q_{3}$ | 0.758(0.756-0.760)  0.851(0.850-0.851)  0.971(0.962-0.981) | 0.707(0.700-0.714)  0.781(0.772-0.790)  0.814(0.809-0.819) | 0.660(0.656-0.664)  0.846(0.844-0.849)  0.846(0.843-0.848) | 0.787(0.782-0.793)  0.870(0.862-0.879)  0.902(0.901-0.904) |
| $\delta$ | $\delta_{1}$  $\delta_{2}$  $\delta_{3}$ | 0.159(0.156-0.162)  0.213(0.202-0.224)  0.292(0.276-0.307) | 0.180(0.171-0.188)  0.207(0.200-0.214)  0.284(0.274-0.293) | 0.173(0.165-0.181)  0.274(0.260-0.289)  0.375(0.356-0.396) | 0.293(0.288-0.298)  0.264(0.240-0.288)  0.362(0.329-0.395) |
| $\delta_{q}$ |  | 0.433(0.415-0.451) | 0.507(0.481-0.533) | 0.708(0.670-0.747) | 0.749(0.718-0.781) |
| $\gamma$ |  | 1/7 | 1/7 | 1/7 | 1/7 |
| $\gamma_{H}$ |  | 1/7 | 1/7 | 1/7 | 1/7 |
| $d$ |  | 4.507(4.204-4.809)*e-5 | 4.613(4.376-4.850)*e-5 | 4.380(4..254-4.506)*e-5 | 3.434(3.212-3.655)*e-5 |
| **Initial value** |  | **Value(95%CI)** | | | |
|  |  | **Songjiang** | **Qingpu** | **Fengxian** | **Chongming** |
| $S(0)$ |  | 1909700 | 1271400 | 1140900 | 637900 |
| $E(0)$ |  | 4.856(4.775-4.936) | 6.218(5.674-6.763) | 7.327(7.102-7.552) | 7.288(5.802-8.774) |
| $I(0)$ |  | 0 | 0 | 0 | 3 |
| $S_{q}(0)$ |  | 1 | 0 | 0 | 10 |
| $E_{q}(0)$ |  | 0 | 0 | 0 | 0 |
| $I_{q}(0)$ |  | 6 | 3 | 2 | 0 |
| $H(0)$ |  | 0 | 0 | 0 | 0 |
| $R(0)$ |  | 0 | 0 | 0 | 0 |
| $D(0)$ |  | 0 | 0 | 0 | 0 |

**Table S5** Basic calculations for 16 districts in Shanghai

|  | Population density (persons/${km}^{2}$) | Date for daily number of reported cases to fall below or equal to 5 |
| --- | --- | --- |
| **Center-region group**  **(average)** | **18756** | **5 June** |
| Huangpu | 40500 | 7 June |
| Hongkou | 30870 | 11 June |
| Jing’an | 24865 | 30 May |
| Yangpu | 17541 | 5 June |
| Xuhui | 16727 | 15 June |
| Putuo | 16182 | 4 June |
| Changning | 15263 | 6 June |
| Baoshan | 3727 | 26 May |
| Minhang | 3127 | 2 June |
| Pudong New Area | 2537 | 7 June |
| **Boundary-region group**  **(average)** | **914** | **17 May** |
| Jiading | 1401 | 11 May |
| Songjiang | 1089 | 14 May |
| Jinshan | 887 | 17 May |
| Fengxian | 786 | 24 May |
| Qingpu | 746 | 21 May |
| Chongming | 574 | 12 May |

**Table S6** Value of parameters and variables for boundary and central region groups

| **Parameter** | | **Value(95%CI)** | |
| --- | --- | --- | --- |
|  |  | **Central-region group** | **Boundary-region group** |
| $c$ | $c_{1}$  $c_{2}$  $c_{3}$  $c_{4}$  $k$ | 31.230(31.177-31.283)  14.128(14.108-14.148)  6.885(6.874-6.897)  8.498(8.483-8.512)  0.310(0.304-0.316) | 27.230(27.161-27.300)  12.124(12.100-12.150)  6.583(6.566-6.600)  8.596(8.574-8.618)  0.309(0.300-0.318) |
| $\beta$ |  | 0.0780(0.0764-0.0797) | 0.071(0.075-0.080) |
| $\beta_{q}$ |  | 0.585(0.571-0.600) | 0.592(0.571-0.613) |
| $\lambda$ |  | 1/14 | 1/14 |
| $\sigma$ |  | 1/3 | 1/3 |
| $q$ | $q_{1}$  $q_{2}$  $q_{3}$ | 0.610(0.599-0.620)  0.796(0.777-0.809)  0.700(0.689-0.710) | 0.632(0.615-0.648)  0.821(0.793-0.843)  0.710(0.693-0.725) |
| $\delta$ | $\delta_{1}$  $\delta_{2}$  $\delta_{3}$ | 0.182(0.178-0.186)  0.232(0.228-0.236)  ${1.3\times\delta}_{2}$ | 0.152(0.147-0.157)  0.242(0.236-0.249)  ${1.3\times\delta}_{2}$ |
| $\delta_{q}$ |  | 0.477(0.468-0.486) | 0.489(0.476-0.504) |
| $\gamma$ |  | 1/7 | 1/7 |
| $\gamma_{H}$ |  | 1/7 | 1/7 |
| $d$ |  | 3.004(3.000-3.008)*e-5 | 3.004(3.000-3.008)*e-5 |
| **Initial value** | | **Value (95%CI)** | |
|  |  | **Central-region group** | **Boundary-region group** |
| $S(0)$ |  | 11572400 | 6979100 |
| $E(0)$ |  | 10.431(10.414-10.448) | 9.431(9.408-9.455) |
| $I(0)$ |  | 0 | 0 |
| $S_{q}(0)$ |  | 1 | 1 |
| $E_{q}(0)$ |  | 0 | 0 |
| $I_{q}(0)$ |  | 1 | 4 |
| $H(0)$ |  | 0 | 0 |
| $R(0)$ |  | 0 | 0 |
| $D(0)$ |  | 0 | 0 |

**Table S7** Peak time, end time, final epidemic size in the retrospective analysis of SI Fig. 3.

| Advanced days | 0 | 3 | 5 | 7 | 10 | 14 |
| --- | --- | --- | --- | --- | --- | --- |
|  | | | | | | |
| Peak time | 46 | 43 | 41 | 39 | 36 | 32 |
| End time | 122 | 114 | 109 | 104 | 96 | 85 |
| Final epidemic size | 625516 | 306159 | 119949 | 117767 | 57429 | 21971 |

**Reference**

[1] Wang, Hao et al. Home quarantine or centralized quarantine? A mathematical modelling study on the COVID-19 epidemic in Guangzhou in 2021. Mathematical biosciences and engineering : MBE, 2022, 9(19): 9060-9078.

[2] Green, Will et al. Inferring the reproduction number using the renewal equation in heterogeneous epidemics. Journal of the Royal Society Interface, 2022(TN.188), 19.

[3] Hinch, Robert et al. Estimating SARS-CoV-2 variant fitness and the impact of interventions in England using statistical and geo-spatial agent-based models. Philosophical transactions. Series A, Mathematical, physical, and engineering sciences, 2022, 380(2233).

[4] Azmon, Amin et al. On the estimation of the reproduction number based on misreported epidemic data. Statistics in Medicine, 2014, 33(7):1176-1192.

[5] Zhou, Weike et al. The resurgence risk of COVID-19 in China in the presence of immunity waning and ADE: A mathematical modelling study. Vaccine, 2022, 40: 7141-7150.

[6] S. Abbott, J. Hellewell, R. N. Thompson et al., Estimating the time-varying reproduction number ofSARS-CoV-2 using national and subnational case counts, Wellcome Open Research, 2020, 5(112), 112.

[7] A. Cori, N. M. Ferguson, C. Fraser and S. Cauchemez, A new framework and software to estimate time-varying reproduction numbers during epidemics, Am. J. Epidemiol., 2013, 178(9), 1505–1512.

[8] WeChat Platform of Shanghai Municipal People's Government Information Office. The free population and the quarantined population. 2022. <https://act-shfb.ibbtv.cn/jiayoushanghai/ym/yq.html>. Accessed 1 May 2022.
